# Phospholipids and peroxisomes in ferroptosis: the therapeutic target of acupuncture regulating vascular cognitive impairment and dementia

**DOI:** 10.3389/fnagi.2025.1512980

**Published:** 2025-04-29

**Authors:** Wenyu Zhang, Ruiyu Li, Donglei Lu, Xinliang Wang, Qiuxuan Wang, Xuyang Feng, Sai Qi, Xuezhu Zhang

**Affiliations:** ^1^First Teaching Hospital of Tianjin University of Traditional Chinese Medicine, Tianjin, China; ^2^National Clinical Research Center for Chinese Medicine Acupuncture and Moxibustion, Tianjin, China; ^3^Tianjin University of Traditional Chinese Medicine, Tianjin, China; ^4^Beijing University of Chinese Medicine Shenzhen Hospital (Longgang), Shenzhen, China; ^5^Sports Training Academy of Tianjin University of Sport, Tianjin, China

**Keywords:** acupuncture, VICD, ferroptosis, peroxisome, PUFA, plasmalogen, lipid peroxidation

## Abstract

Ferroptosis, since its conceptualization in 2012, has witnessed an exponential growth in research interest over recent years. It is regulated by various cellular metabolic pathways during chronic cerebral ischemia and hypoxia, including reactive oxygen species (ROS) generation, iron accumulation, abnormalities in glutathione metabolism, and disruptions in lipid and glucose metabolism. With the deepening and widespread research, ferroptosis has emerged as a critical pathway in the pathogenesis of vascular cognitive impairment and dementia (VCID). This unique cell death pathway caused by iron-dependent phospholipid peroxidation is strongly related to VICD. We examine the impact of phospholipid composition on neuronal susceptibility to ferroptosis, with a particular focus on the critical role of polyunsaturated fatty acids (PUFAs) in this process. Intriguingly, peroxisomes, as key regulators of lipid metabolism and oxidative stress, influence the susceptibility of neuronal cells to ferroptosis through the synthesis of plasmalogens and other lipid species. In this Review, we provide a critical analysis of the current molecular mechanisms and regulatory networks of acupuncture for ferroptosis, the potential functions of acupuncture in peroxisomal functions and phospholipid metabolism, and its neuroprotective effects in VCID, together with a potential for therapeutic targeting. As such, this highlights the theoretical basis for the application of acupuncture in VCID through multi-target regulation of ferroptosis. This review underscores the potential of acupuncture as a non-pharmacological therapeutic approach in VCID, offering new insights into its role in modulating ferroptosis and associated metabolic pathways for neuroprotection.

## 1 Introduction

Ferroptosis is a form of cell death characterized by intracellular iron overload, increased production of cell membrane phospholipid peroxides, and decreased antioxidant scavenging capacity, leading to cell membrane perforation and death (Stockwell, 2022). Various molecules within cells affect intracellular iron levels, and lipid peroxidation status regulates the occurrence of ferroptosis (Chen et al., 2021b). After cells undergo ferroptosis, they will show signs that it has more unique morphological characteristics compared with other forms of death such as apoptosis (Galluzzi et al., 2018), autophagy (Hou et al., 2016), and necrosis (Tang et al., 2019). Some key features of ferroptosis include mitochondrial shrinkage, increased mitochondrial membrane density and potential, reduction or loss of mitochondrial cristae, and rupture of the outer mitochondrial membrane (Dixon et al., 2012). Multiple studies have demonstrated that ferroptosis plays a significant role in the onset and progression of various diseases, including neurological disorders (Ou et al., 2022), renal diseases (Belavgeni et al., 2020), diabetes, cardiovascular diseases (Fang et al., 2023), and more. Nevertheless, ferroptosis plays a more important role in the pathological process of VCID and deserves further exploration.

Peroxisomes, which are membrane-enclosed organelles involved in oxidative processes, play crucial roles in cellular lipid metabolism in processes such as the breakdown of very-long-chain fatty acids and the catabolism of hydrogen peroxide by catalase (CAT) (Wanders et al., 2023). Furthermore, upon activation, peroxisomes can increase the sensitivity of cells to ferroptosis (Zou et al., 2020a). Zou et al. (2020a) recently published their research in *Nature*, demonstrating that peroxisomes play a critical role in ferroptosis by producing plasmalogens, which serve as substrates for lipid peroxidation. Plasmalogens are more prone to oxidation than other lipid components, and peroxisomes are the sole organelles responsible for their biosynthesis (Zou et al., 2020a). This means that peroxisomes are involved in regulating the sensitivity of cells to ferroptosis. These discoveries offer a novel understanding of the lipid metabolic foundation of ferroptotic cell death.

Existing studies suggest that ferroptosis may mediate the physiological and pathological mechanisms of VCID (Yan and Zhang, 2019). Lipid peroxidation, a hallmark of ferroptosis, is closely linked to the severe lipid metabolism disorders observed in VCID patients (Gustaw-Rothenberg et al., 2010). Moreover, metabolomic analyses have revealed significant alterations in various cell membrane phospholipids, including sphingomyelin (SM), phosphatidylcholine (PC), and phosphatidylethanolamine (PE) in VCID patients (Ma, 2023). The composition of these phospholipids determines cellular sensitivity to ferroptosis. Acyl-CoA synthetase long-chain family member 4 (ACSL4) catalyzes the esterification of free fatty acids into CoA in an ATP-dependent manner, making PE species containing arachidonic acid (AA) and adrenic acid (AdA) the preferred substrates for oxidation (Cao et al., 2000). Thus, ACSL4 increases sensitivity to ferroptosis by specifically esterifying AA and AdA into PE. Cerebral ischemia and hippocampal lesions has been identified as the primary pathological mechanisms underlying the development of VCID. Studies have shown that (Gubern et al., 2013) ACSL4 is widely expressed in the brains of rats with focal ischemia, particularly in the hippocampal CA1 region, and is closely associated with the development of both VCID and ferroptosis. Furthermore, iron gradually accumulates in ischemic and hypoxic brain tissue, and neuronal iron deposition is closely associated with neurodegeneration and cognitive impairment (Li et al., 2012). The most serious neuronal death occurred in the CA1 where the most iron deposits were observed (Du S.-Q. et al., 2017). An increasing number of studies suggest that ferroptosis may be an effective therapeutic target for VCID intervention (Ratan, 2020; Weiland et al., 2019). Animal studies have confirmed the presence of ferroptosis in the brains of vascular dementia (VD) rats (Huo et al., 2019), and many of these pathological changes can be modulated by acupuncture (Wang G.-L. et al., 2023). Therefore, this article reviews the current mechanisms and related research on ferroptosis in VICD. We have focused on summarizing the latest progress and potential therapeutic targets of acupuncture methods in ferroptosis in VICD and have innovatively summarized the important roles played by peroxisomes and phospholipid metabolism, as well as the possible intervention targets of acupuncture. This may provide innovative ideas for further VICD research.

## 2 The characteristic and epidemiology of VCID

**FIGURE 1:**
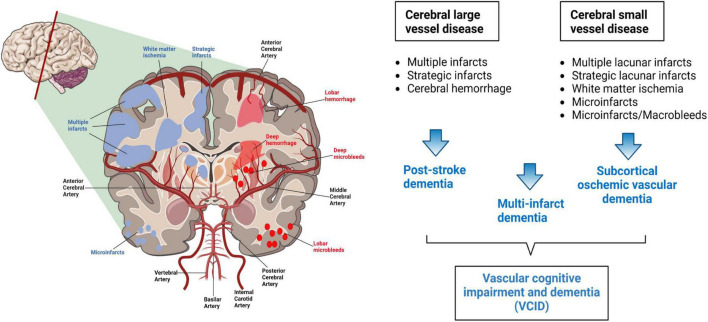
The classification of VCID.

VCID result from multiple types of cerebrovascular injury, including large vessel stroke and microvascular dysfunction (Inoue et al., 2023). The Vascular Impairment of Cognition Classification Consensus Study (VICCCS) identifies four major subtypes of vascular lesions that cause dementia: (1) post-stroke dementia, (2) subcortical ischemic vascular dementia, (3) multi-infarct dementia, and (4) mixed dementia (Skrobot et al., 2017; Figure 1). Among all types of cerebrovascular events, ischemic cerebrovascular disease is the primary cause of VCID (Yu et al., 2022). Years before the onset of cognitive decline, VCID patients have diffuse decreases in cerebral blood flow in the frontal lobe, hippocampus, and other brain regions (Chang Wong and Chang Chui, 2022).

The prevalence of VCID is significant, and the incidence rate is climbing annually. In North America and Europe, VCID accounts for approximately 15% to 20% of clinically diagnosed dementia cases (Gorelick et al., 2011), while in Asia and some developing countries, the estimated burden of VCID is thought to be closer to 30% (Chan et al., 2013; Jhoo et al., 2008). Approximately 10% of patients develop dementia after their first stroke (Pendlebury and Rothwell, 2009). It results in cognitive and personality abnormalities that have a major negative impact on the patient’s independence in day-to-day activities as well as the family’s overall quality of life (Rundek et al., 2022). Nonetheless, early therapy for VCID is reversible and can lower the patient’s disability rate when compared to other refractory dementias (Iadecola et al., 2019). Therefore, there is substantial clinical and societal value to actively exploring the pathogenesis of VCID and looking for efficient therapies.

## 3 Ferroptosis is closely related to VICD

Ferroptosis is an iron-dependent, lipid-peroxidation-mediated form of cell death (Jiang et al., 2021). At its core, it is a disorder of intracellular metabolic pathways (Liang et al., 2022). Overloading on Fe^2+^ within cells may trigger Fenton reactions, producing a large amount of ROS and directly causing lipid peroxidation. Furthermore, excess Fe^2+^ can act as a cofactor for lipid peroxidation enzymes, increasing lipid peroxidation, disrupting cell membrane phospholipids (mPLs), and triggering ferroptosis (Zou et al., 2020a; Zou et al., 2020b). Antioxidant systems like reduced glutathione (GSH) (Ursini and Maiorino, 2020) and glutathione peroxidase 4 (GPX4) (Liu et al., 2023; Zhang W. et al., 2024) help protect the membrane. When the antioxidant system activity decreases in cells, it is not enough to clear excess lipid peroxides, and eventually the cells die due to membrane perforation (Chen et al., 2021c).

**FIGURE 2:**
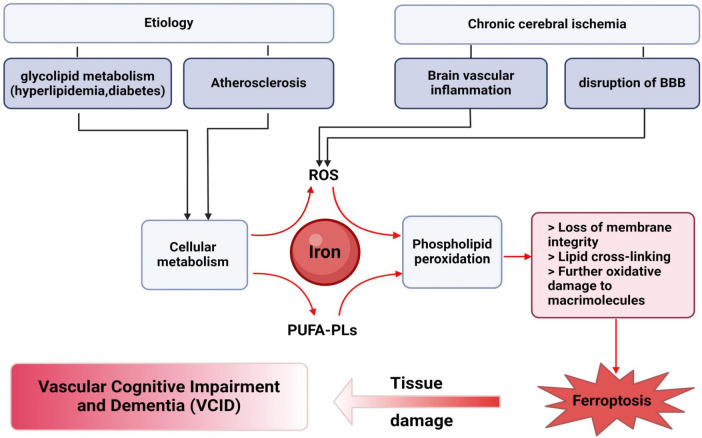
Ferroptosis is closely related to VICD. The pathogenic factors of VICD include abnormal glucose and lipid metabolism, hypertension, diabetes, and atherosclerosis. The above etiology of VICD can lead to metabolic abnormalities in brain cells. Chronic cerebral ischemia is the pathological basis of VICD. Long term inadequate blood flow can lead to the occurrence of cerebrovascular inflammation, damage to the blood-brain barrier, and increased iron ions, collectively resulting in the production of a large amount of ROS in the brain. The occurrence of iron death in VICD is carried out through phospholipid peroxidation under the above conditions, which depends on transition metal iron, ROS and phospholipids containing polyunsaturated fatty acid chains (PUFA-PLs). The occurrence of ferroptosis is unfortunate for VICD, which means that brain neuron cells will face disadvantages, and this may include loss of membrane integrity, disruption of membrane properties through lipid cross-linking, as well as oxidative damage to macromolecules and cellular structures caused by ROS derived from PUFA-PL.

The etiology of VICD is complex, and its pathogenesis has not yet been fully elucidated. In recent years, it has been discovered that ferroptosis is inextricably linked to VCID (Jiang et al., 2021; Li et al., 2018; Figure 2).

### 3.1 Etiology correlation

In terms of etiology, risk factors for VICD such as hyperlipidemia (Appleton et al., 2017; Jia et al., 2020), diabetes (Edgerton-Fulton and Ergul, 2022; Prajjwal et al., 2023), atherosclerosis (Huang et al., 2021), and other diseases are characterized by intracellular metabolic pathways disorders and increased oxidative damage, which are associated with ferroptosis (Figure 2).

Hyperlipidemia is regarded as an independent risk factor for atherosclerosis, which can accelerate the pathogenesis of VCID (Jia et al., 2020) and is also a necessary condition for ferroptosis (Chen Z. et al., 2023). There is an inevitable intrinsic link between VCID and hyperlipidemia (Pan et al., 2024). Statistical studies have found that 41% of VICD patients also have hyperlipidemia (Lüders et al., 2012). Both high levels of low-density lipoprotein (LDL) cholesterol and low levels of high-density lipoprotein (HDL) cholesterol are known risk factors for carotid atherosclerosis and coronary artery disease (Reitz et al., 2004; Sharrett et al., 1994), which may result in cognitive impairment secondary to cerebral hypoperfusion or embolism (Breteler et al., 1994). Dyslipidemia has been found to interact with chronic cerebral hypoperfusion to promote inflammation resulting in cognitive dysfunction in the brain, which may subsequently cause VICD (Shang et al., 2024). Notably, the increase in cholesterol in hyperlipidemia may be associated with ferroptosis in VCID patients (Yan and Zhang, 2019). Cholesterol, as an essential lipid component of mammalian cell membranes, is crucial for maintaining membrane integrity, fluidity, and microstructural organization (Luo et al., 2020). The mevalonate pathway is the primary route for cholesterol synthesis and is one of the critical pathways in cellular metabolism (Juarez and Fruman, 2021). This pathway influences ferroptosis in three distinct ways, including through the regulation of GPX4 (Seibt et al., 2019), squalene, and coenzyme Q10 (CoQ10) (Sun et al., 2023). Studies have shown that statins, by inhibiting the rate-limiting enzyme of the mevalonate pathway, can downregulate GPX4, making hepatic stellate cells more susceptible to ferroptosis (Kitsugi et al., 2023). This indicates that elevated cholesterol levels in the blood of VCID patients may negatively regulate GPX4 activity through the mevalonate pathway, playing a role in promoting cellular ferroptosis. Additionally, 7-dehydrocholesterol (7-DHC), a major metabolite of cholesterol, has been identified as a potential regulator of lipid peroxidation and ferroptosis (Freitas et al., 2024). Using genome-wide clustered regularly interspaced short palindromic repeats- associated protein 9 (CRISPR-Cas9) screening, researchers identified that enzymes involved in distal cholesterol biosynthesis play pivotal roles in regulating ferroptosis by modulating the levels of 7-DHC. This intermediate metabolite is synthesized by sterol C5-desaturase (SC5D) and further metabolized by 7-DHC reductase (DHCR7) for cholesterol synthesis. Research suggests that 7-DHC dictates ferroptosis surveillance by using the conjugated diene to exert its anti-phospholipid autoxidation function and shields plasma and mitochondria membranes from phospholipid autoxidation (Li Y. et al., 2024). It is speculated that VICD patients with hyperlipidemia may have disrupted lipid metabolism in their cells, which may affect the normal metabolism of cholesterol and subsequently impact the synthesis of 7-DHC, thereby affecting their sensitivity to ferroptosis. Furthermore, studies have shown that high-fat diet levels can affect the architecture of brain cell tissues, promote iron accumulation, limit the activity of endogenous antioxidant mechanisms, and may induce ferroptosis in cells (Chang et al., 2023). Therefore, it is speculated that VICD patients with hyperlipidemia, combined with their high-fat dietary characteristics, are more prone to ferroptosis.

Diabetes has been strongly linked to ferroptosis (Liu et al., 2024) and VCID (Biessels and Despa, 2018), as revealed by large-scale epidemiological investigations. Evidence suggests that diabetic patients have a 2.27-fold higher risk of developing vascular dementia compared to non-diabetic individuals (Biessels and Despa, 2018). In fact, it is not only clinically diagnosed diabetes that poses a threat, even elevated blood glucose levels alone can have detrimental effects on the nervous system (Faber et al., 2020). Research has shown that VICD patients with type 2 diabetes (T2D) are more susceptible to ferroptosis (Sha et al., 2021), which further exacerbates neurological dysfunction and worsens cognitive impairment (Luo et al., 2022). A study investigating the relationship between energy metabolism, ferroptosis, and cognitive impairment in the brains of T2D mice revealed that ferroptosis in hippocampal neurons contributes to cognitive dysfunction (Xie, 2023). The use of the ferroptosis inhibitor liproxstatin-1 was found to inhibit ferroptosis and alleviate T2D-related cognitive impairment (Xie, 2023). Furthermore, GPX4 enzyme levels are markedly lower in diabetic patients compared to non-diabetic individuals (Luo et al., 2021). This reduction may cause lipid peroxidation to rise and glutathione levels to fall, which would cause ferroptosis. Previous studies have suggested that the etiology of cognitive impairment and ferroptosis in T2D patients is likely multifactorial. Emerging evidence indicates that it is particularly associated with iron overload in the brain, especially in the hippocampus (Liu et al., 2020). The underlying mechanism may involve high levels of insulin in the brain promoting the redistribution of transferrin receptors (TfR) to the cell surface, thereby increasing iron uptake and leading to neuronal iron overload (Tanner and Lienhard, 1987). Additionally, reports have indicated that serum hepcidin levels are low in T2DM patients (Altamura et al., 2017). Liu et al. (2020) found that hippocampal iron deposition in T2DM rats is closely related to the expression of hepcidin, and its deficiency is a key factor contributing to systemic iron overload.

Atherosclerotic lesions form prior to the clinical symptoms and signs of VCID (Huang et al., 2021), and the crosstalk between atherosclerosis and hyperlipidemia disrupts lipid metabolism in VCID patients. This disruption is one of the key factors inducing ferroptosis (Yang et al., 2022c). Oxidized low-density lipoprotein (Ox-LDL) is not only a key initiator of atherosclerosis but also a driving force behind atherosclerosis progression (Pirillo et al., 2013). Ox-LDL contributes to endothelial cell injury, promotes inflammatory responses, stimulates smooth muscle cell proliferation, and induces cellular ferroptosis (Yu et al., 2023). Bai et al. (2020) treated normal aortic endothelial cells (NAECs) from ApoE^–/–^ mice fed a high-fat diet (HFD) with ox-LDL and erastin, respectively, and found significantly increased levels of intracellular ROS, lipid peroxides, and malondialdehyde (MDA), indicating that ox-LDL, similar to erastin, can induce ferroptosis in NAECs. Additionally, ox-LDL treatment of human coronary artery endothelial cells (HCAEC) led to the accumulation of lipid peroxides and elevated ROS levels, further promoting ferroptosis in HCAECs (He et al., 2021; Yang et al., 2021). The above evidence suggests that atherosclerosis may influence vascular endothelial cells in VCID patients through ox-LDL, leading to ferroptosis. In addition, vascular stenosis caused by carotid atherosclerosis can put the brain in a long-term hypoperfusion state, resulting in chronic ischemia, hypoxia, and material exchange obstacles in brain tissue (Matsumoto et al., 2018). At the same time, oxidative stress in oligodendrocytes and microglia rises, resulting in reactive oxygen species that cause lipid peroxidation, may induce ferroptosis in neuronal cells, destroy neuronal structure, and lead to vascular dementia (Abe et al., 2024; Liu S. et al., 2022; Wang H. et al., 2024).

### 3.2 Pathological manifestations correlation

The occurrence of ferroptosis first requires the presence of iron ions (Tang et al., 2021). Excessive iron accumulation in the tissue, particularly in the form of unstable ferrous ions, can react with H_2_O_2_ within cells through Fenton and Haber-Weiss reactions (Henning et al., 2022). Hydroxyl radicals are extremely potent oxidizing agents that rapidly react with lipid molecules in cell membranes, initiating lipid peroxidation. Polyunsaturated fatty acids (PUFAs) in the cell membrane are the primary targets of hydroxyl radicals, ultimately leading to membrane structural damage. Additionally, the breakdown of lipid peroxides generates other harmful secondary metabolites, such as MDA and 4-hydroxynonenal (4-HNE), which further damage membrane proteins and DNA (Long et al., 2023). Ultimately, this process leads to the ferroptosis of neurones due to iron overload (Kenny et al., 2019). Clinical and experimental studies show that throughout the pathological development of VICD, there are multiple manifestations of iron accumulation that are intimately associated to the occurrence of ferroptosis (Moon et al., 2016; Sun et al., 2017).

Because getting pathological sections from clinical patients is highly difficult, magnetic resonance imaging (MRI) technology is now mostly employed to research brain iron accumulation in clinical settings (Chaudhary et al., 2019). Common techniques include lateral relaxation rate (R2), apparent relaxation rate (R2*) (Sethi et al., 2022), effective lateral relaxation time (T2*) (McNeill et al., 2008), susceptibility weighted imaging (SWI) (Kirsch et al., 2009; Sotoudeh et al., 2021), and quantitative susceptibility mapping (QSM) (Madden and Merenstein, 2023; Spence et al., 2022).

Unlike hemosiderin deposition induced by intracranial small vessel injury in microbleeds, cerebral iron accumulation is caused by an excess deposition of free iron ions in nerve cells due to iron dyshomeostasis problems (DeGregorio-Rocasolano et al., 2019). Clinical studies have confirmed that in VCID patients, iron accumulation is widely present on the cerebral cortex and subcortical related areas (Mendes et al., 2020), such as the hippocampus and putamen, and the level of iron accumulation may be related to the severity of cognitive impairment (Liu et al., 2015). Furthermore, a clinical study evaluated cerebral iron accumulation using a quantitative susceptibility map and found that the higher content of cerebral iron accumulation, the more likely patients with vascular risk factors or cerebrovascular events are to develop VCID (Li M. et al., 2021).

QSM can achieve a non-invasive quantitative evaluation of brain iron accumulation content through the inversion relationship between field maps and magnetic susceptibility (Vinayagamani et al., 2021). Researchers investigated the iron accumulation in the brains of VCID patients caused by subcortical ischemia using QSM technology and discovered that the iron accumulation is widespread in the patients’ brains and is closely related to damage in different cognitive areas (Sun et al., 2017). Also, the most recent study used QSM technology to investigate the link between iron accumulation and cognitive function in high blood pressure patients. It found that there was a lot more iron in deep brain regions like the caudate nucleus and putamen in high blood pressure patients who had cognitive impairment and a link with cognitive performance (Qin et al., 2022). This suggests that iron deposition abnormalities can occur before cerebrovascular events occur, highlighting the importance of early intervention for iron deposition in VCI patients.

According to the research, iron accumulation in the brain of VCID patients occurs mostly in the basal ganglia and frontal cortex, which are the common sites of ischemic stroke (Jiang, 2020). The increase in iron is associated with a decrease in T2 relaxation time in MRI, resulting in a hypointense signal in T2-weighted images (Brass et al., 2006). In patients with cortical ischemia at the early clinical stage, T2- and proton density-weighted MRI revealed a decrease in intensity in the subcortex, indicating iron accumulation in the region (Ida et al., 1994). In patients with ischemic stroke (Ogawa et al., 1997; van Etten et al., 2015) or anoxic-ischemic injury (Dietrich and Bradley, 1988), T2-weighted MRI detects a low-intensity signal in the thalamus or basal ganglia ipsilateral to the infarction that is distant from the ischemic zone and is associated with poor cognitive function and negative emotion (Kuchcinski et al., 2017). VCI is the initial stage of VCID development. Imaging studies have revealed that VCI patients exhibit widespread microstructural abnormalities in cerebral white matter along with increased iron deposition. This rise in cerebral white matter iron deposition is significantly correlated with compromised brain myelin integrity. Furthermore, VCI patients show significantly elevated serum levels of white matter myelin injury markers. The levels of ferroptosis-related indicators in the serum, such as ACSL4, GPX4, and GSH, are also significantly associated with myelin injury markers. These findings collectively suggest that ferroptosis may play a crucial role in the occurrence and progression of VCID (Gu, 2023).

Apart from the clinical results, studies on animals and cell cultures have also produced evidence that connects iron to ischemic neuronal damage and VCID (Wu et al., 2018). Researchers established the VICD rat model by performing permanent bilateral carotid artery blockage surgery to evaluate iron and oxidative stress indicators (Li et al., 2012). The study found that iron accumulation was observed in both the hippocampal CA1 region and cerebral cortex, which is associated with local neuronal death and increased lipid peroxidation. Research has confirmed that the majority of ischemic stroke will develop into VCID (Inoue et al., 2023) and the middle cerebral artery occlusion (MCAO) rodent model is the most commonly used animal model for creating ischemic stroke (Yang et al., 2016). There is a considerable rise in the amounts of free iron or ferritin in the brains of mice, rats, and gerbils with MCAO (Chi et al., 2000; Ding et al., 2011; Fang et al., 2013; Tuo et al., 2017). After 4 weeks of transient forebrain ischemia caused by the four-vessel occlusion model, iron deposition was found in the rat brain (Kondo et al., 1995). The photoactivation of photosensitive dyes transmitted to the bloodstream can also cause occlusion in rats, and the level of free iron increases 1 h after occlusion (Millerot-Serrurot et al., 2008). Three weeks after middle cerebral artery occlusion/reperfusion (MCAO/R) surgery, iron deposition was also observed in the thalamus of rats, accompanied by a low signal detected by T2 weighted MRI (Justicia et al., 2008), consistent with observations in the human brain. Iron supplementation significantly increased the cerebral infarction volume in rats 24 h after MCAO/R (Mehta et al., 2004). When the iron chelating drug deferoxamine (DFO) is given nasally prior to or following occlusion, it can considerably reduce infarct size (Hanson et al., 2009). Additionally, a different iron chelator called 2,2′-bipyridine has been demonstrated to effectively decrease the size of the infarct in the rats with cortical photothrombotic vascular occlusion (Demougeot et al., 2004).

Recent advancements in single-cell analysis, such as single-cell RNA sequencing (scRNA-seq) and spatial transcriptomics (Milenkovic et al., 2024), offer unprecedented opportunities to study ferroptosis-related mechanisms in VCID with high resolution and specificity. These technologies enable a more precise understanding of how ferroptosis contributes to neurovascular dysfunction, cognitive decline, and treatment responses in VCID patients. For instance, scRNA-seq allows for cell-specific analysis of ferroptosis markers, facilitating the identification of ferroptosis-prone cell populations—such as neurons, astrocytes, microglia, and endothelial cells—in VCID-affected regions like the hippocampus and white matter (Dang et al., 2022). While direct studies linking single-cell sequencing to ferroptosis in VCID are currently limited, research in related neurological conditions provides valuable insights. For example, a single-cell and spatial transcriptomics study based on the ICH rat model revealed that ferroptosis is a primary form of programmed cell death following intracerebral hemorrhage, predominantly affecting mature oligodendrocytes (Gu et al., 2024). This process was observed as early as 1 h post-hemorrhage, peaking at 24 h, and was notably present in regions such as the hippocampus and choroid plexus. These findings underscore the potential of single-cell technologies to elucidate cell-type-specific ferroptosis dynamics in brain disorders, which could be applicable to VCID research (Pozojevic and Spielmann, 2023; Yan and Zhang, 2019).

## 4 Comparative analysis of ferroptosis in VCID, AD, and PD

Ferroptosis is emerging as a shared mechanism of neuronal death in multiple neurodegenerative diseases, including VCID, Alzheimer’s disease (AD), and Parkinson’s disease (PD) (Fei and Ding, 2024; Reichert et al., 2020; Yan H. et al., 2021). However, the specific triggers, affected brain regions, and metabolic dysregulations associated with ferroptosis differ among these disorders. Understanding these distinctions is critical for designing disease-specific ferroptosis-targeting therapies.

In VCID, chronic cerebral hypoperfusion leads to blood-brain barrier (BBB) dysfunction and ischemia-induced metabolic stress, resulting in iron accumulation in ischemic hippocampal, white matter lesions and deep gray matter structures, which exacerbates oxidative damage and promotes ferroptosis (Gao et al., 2025). In contrast, iron overload in AD is commonly observed in hippocampal and cortical regions, where it is closely associated with amyloid-beta (Aβ) aggregation and tau hyperphosphorylation, catalyzing lipid peroxidation and oxidative stress (Majerníková et al., 2024). Thus, both diseases share hippocampal iron accumulation as a common pathological feature. Similarly, PD exhibits ferroptosis susceptibility due to iron accumulation in the substantia nigra, where dopaminergic neurons are highly vulnerable to iron-catalyzed lipid peroxidation, exacerbated by dopamine metabolism-induced ROS production (Levi et al., 2024).

Lipid peroxidation further contributes to ferroptotic damage in these conditions. VCID-related ischemia disrupts plasmalogen metabolism and peroxisomal function, accelerating the peroxidation of PUFAs in synaptic membranes, with GPX4 depletion and lipoxygenase (ALOX) activation heightening ferroptosis susceptibility (Han et al., 2024). In AD, oxidative stress increases susceptibility to ferroptotic lipid peroxidation and elevating levels of oxidized phosphatidylethanolamines (oxPEs) and 4-hydroxynonenal (4-HNE) (Chen et al., 2021a). NADPH oxidase 4 (NOX4), which is a major source of ROS, induces astrocyte ferroptosis accompanied by impaired mitochondrial metabolism by reducing five protein complexes in the astrocyte mitochondrial electron transport chain, NOX4 promotes oxidative stress-induced lipid peroxidation accompanied by the increased expression of the markers 4-HNE and MDA. Park et al. (2021) showed that NOX4 promotes the ferroptosis of astrocytes by triggering oxidative stress-induced lipid peroxidation via impaired mitochondrial metabolism in AD. In PD, dopaminergic neurons, rich in PUFAs, experience extensive oxidative lipid damage, where dopamine metabolism itself generates ROS, fostering a ferroptotic environment, and ultimately lead to the degeneration of dopaminergic neurons (Ding X.-S. et al., 2023). Microglial activation and M1 polarization are related to Gpx4 and GSH content reduction and lipid peroxidation, all of which are involved in ferroptosis. Hou et al. (2019) revealed that neurodegeneration of the LC/NE system plays a critical role in mediating learning and memory dysfunction in a two pesticide-induced mouse model of PD and that this role was mediated through ferroptosis and microglia-mediated neuroinflammation.

Given these disease-specific ferroptotic mechanisms, therapeutic approaches must be tailored accordingly. In VCID, targeting iron homeostasis, peroxisomal metabolism, and lipid peroxidation represents a key strategy. Acupuncture has demonstrated potential in modulating iron homeostasis, reducing oxidative stress, and enhancing cerebral perfusion, making it a promising ferroptosis-regulating intervention (Li M.-Y. et al., 2022; Liang et al., 2021). AD-related therapies focus on iron chelators (e.g., deferiprone), GPX4 activators, and omega-3 PUFA supplementation to mitigate excessive lipid peroxidation (Majerníková et al., 2021; Plascencia-Villa and Perry, 2021). In PD, ferroptosis inhibition strategies include iron chelation therapy, Nuclear factor erythroid 2-related factor 2 (Nrf2) pathway activation, and targeting mitochondrial dysfunction, all of which aim to restore redox balance and protect vulnerable dopaminergic neurons (Abdalkader et al., 2018; Zhou et al., 2024).

## 5 Potential sex differences in ferroptosis susceptibility and implications for VCID

Emerging evidence suggests that biological sex could influence ferroptosis susceptibility, potentially contributing to sex-specific differences in VCID risk, progression, and treatment response (Ru et al., 2024). These differences may be associated with hormonal regulation, iron metabolism, lipid composition, and antioxidant defense mechanisms, although further research is needed to confirm these relationships. Males tend to have higher systemic iron levels than females due to the absence of menstrual iron loss, which might predispose them to greater ferroptotic vulnerability and increased iron accumulation in VCID-related brain regions (e.g., hippocampus, deep gray matter) (Allegra et al., 2024). Conversely, postmenopausal women experience a sharp increase in iron stores, which may elevate ferroptosis risk later in life, but this requires further investigation. Some studies suggest that females may have higher PUFA levels in neuronal membranes, which could make them more susceptible to lipid peroxidation-driven ferroptosis (Wang L. et al., 2023). However, the extent to which sex-specific lipid metabolism influences VCID pathology remains unclear. Estrogen has been reported to upregulate antioxidant pathways (e.g., Nrf2-GPX4 axis), potentially providing greater resistance to ferroptosis in premenopausal females (Das et al., 2017). After menopause, the loss of estrogen-mediated protection may lead to increased oxidative stress and ferroptotic damage, which could contribute to higher VCID risk in older women (Ide et al., 2022).

## 6 Potential pathological mechanism targets of acupuncture intervention

In terms of pathological mechanisms, VICD patients experience chronic hypoperfusion of brain tissue, reduced oxygen and nutrient supply, accumulation of harmful substances, as well as abnormalities such as iron metabolism disorder, increased ROS, antioxidant dysfunction, and an inflammatory response (Fu et al., 2023; Sun et al., 2017). It has come to our attention that this is strongly connected to the three fundamental mechanisms of ferroptosis events: iron dyshomeostasis, oxidative damage caused by lipid peroxidation, and imbalance of the cellular antioxidant system (Yan and Zhang, 2019).

### 6.1 The regulatory effects of acupuncture on iron homeostasis

Chronic cerebral ischemia and hypoxia in VICD lead to damage of microvascular endothelial cells and the BBB, resulting in blood extravasation into brain parenchyma and the release of large amounts of Fe^2+^ from ruptured red blood cells. Excessive accumulation of iron during iron metabolism can trigger oxidative stress responses, ultimately leading to lipid peroxidation of cell membranes and cell death. Since VICD occurs after a cerebral stroke and is a result of stroke disease progression, the pathological mechanism of BBB disruption in VICD is consistent with the consequences of BBB damage after stroke. Both conditions can lead to iron overload and ferroptosis. The regulatory effects of acupuncture on both conditions are based on this shared pathological mechanism.

Acupuncture modulates iron metabolism-related molecules and pathways to restore iron homeostasis and reduce the occurrence of ferroptosis. Hepcidin is the primary regulatory factor in iron metabolism within the body. Its main function involves binding to the iron transporter ferroportin (FPN), promoting its degradation, and thereby inhibiting the release of iron from cells into the bloodstream. Studies have shown that acupuncture improves iron homeostasis by regulating hepcidin levels. Experiments indicate that electroacupuncture (EA) can lower neurological function scores in cerebral stroke rats and may exert neuroprotective effects by reducing hepcidin protein and gene expression, thus enhancing brain iron metabolism and decreasing excessive iron accumulation (Chen et al., 2022; Zhang et al., 2023).

Ferroportin 1 (Fpn1) is a beneficial iron export transporter located on the cell membrane, responsible for transferring iron from inside the cell to the outside, thereby regulating iron balance in the body. In contrast, increased expression of transferrin receptor 1 (TfR1) leads to more iron entering the cell, which may result in iron overload, elevated production of ROS, enhanced lipid peroxidation, and ultimately, ferroptosis. Using the Xingnao Kaiqiao acupuncture method to treat rats with cerebral ischemia-reperfusion injury (CIRI), the results suggest that acupuncture can increase levels of Fpn1, GSH and GPX4 in the ischemic brain tissue, decrease the content of MDA and TfR1 proteins, and inhibit ferroptosis in neuronal cells (Wang Q. et al., 2024). Additionally, research (Chen et al., 2022) has shown that EA at Baihui (DU20) and Dazhui (GV14) acupoints significantly decreased the expression of TfR1, transferrin (Tf), and ferritin (Ft) in intracranial hemorrhage (ICH) rat models, promoting brain iron metabolism, restoring iron homeostasis, and providing neuroprotection.

Acupuncture can also regulate the expression of ferritin heavy chain (FTH1), enhance the ability of brain cells to store iron, reduce the accumulation of free iron, and inhibit the occurrence of ferroptosis. Another study (Li M.-Y. et al., 2022) found that stimulating the acupoints DU20 and Qubin (GB7) significantly increased FTH1 expression levels in the brain tissue of ICH rat models. This upregulation helps to increase iron storage capacity, reduce free iron accumulation, prevent its involvement in the Fenton reaction, which produces harmful ROS, and inhibit ferroptosis.

Moreover, acupuncture supports systemic iron homeostasis by improving hepatic and intestinal iron metabolism functions (Ding L. et al., 2023; Gao et al., 2017; Xia et al., 2017; Yu et al., 2024). For instance, acupuncture at the points of Ganshu (BL 18) and Pishu (BL 20) can enhance liver function, while stimulation of Zusanli (ST36) can improve intestinal iron absorption. Furthermore, acupuncture can regulate the key molecules such as heme oxygenase 1 (HO-1) and iron regulatory protein 1 (IRP1) to enhance the antioxidant capacity of brain tissue in VD rats, reducing neuroinflammation caused by ferroptosis (Du S. et al., 2017). Therefore, acupuncture modulates iron homeostasis through multiple pathways and targets, and inhibit ferroptosis.

Pharmacological iron chelators, such as DFO, directly bind free iron to reduce oxidative stress and prevent ferroptosis. However, their long-term use may disrupt systemic iron balance, leading to potential deficiencies and metabolic disturbances (Velasquez and Wray, 2025). In contrast, acupuncture provides a homeostatic regulation of iron metabolism by influencing key molecular pathways rather than indiscriminately depleting iron. Studies suggest that acupuncture downregulates transferrin receptor 1 (TfR1) to limit excessive iron uptake while upregulating FPN1 to facilitate iron efflux, thereby preventing intracellular iron accumulation in postoperative cognitive dysfunction (POCD) mouse model (Chen et al., 2025). Additionally, acupuncture modulates hepcidin levels in iron-deficient obese patients, ensuring a dynamic and balanced distribution of iron without inducing systemic iron depletion (Xie et al., 2017). This indirect but systemic approach may offer a more sustainable neuroprotective strategy against ferroptosis in VCID.

Beyond iron transport regulation, acupuncture enhances cellular antioxidant defenses, complementing its effects on iron homeostasis (Su et al., 2020). Unlike iron chelators, which primarily remove iron but do not address oxidative damage, acupuncture promotes the activity of GPX4, superoxide dismutase (SOD), and CAT, all of which mitigate lipid peroxidation and oxidative stress (Zhao et al., 2022). This dual-action—stabilizing iron homeostasis and reinforcing antioxidant capacity—allows acupuncture to counteract ferroptosis with potentially fewer adverse effects compared to pharmacological chelation (Chen and Hsieh, 2020). Given these advantages, future research should explore whether acupuncture combined with iron chelators could optimize ferroptosis suppression by balancing immediate iron detoxification with long-term metabolic stability.

### 6.2 The regulatory effects of acupuncture on cellular antioxidant system

Chronic cerebral ischemia in VCID not only directly causes the generation of a large amount of ROS within the brain, but also promotes ROS production through inflammatory responses, resulting in neuronal lipid peroxidation damage and the formation of the end product MDA (Wu et al., 2023), exacerbating brain tissue damage in VCID animal models (Yang, 2024). The Fe^2+^ ions that are released when the BBB ruptures could be involved in the Fenton reaction, resulting in the production of a significant quantity of ROS, which in turn directly enhances the process of lipid peroxidation. Additionally, Fe^2+^ can act as a cofactor for lipid peroxidation enzymes, further inducing ferroptosis (Liang, 2023). The deterioration of antioxidant system function weakens the ability of cells to resist lipid peroxidation and promotes the occurrence of ferroptosis. In the brain tissues of patients with ischemic stroke and animal models with MCAO, reduced levels of GSH increase the sensitivity of brain to ferroptosis-induced damage (Wu et al., 2023). Related studies have also shown that upregulating GPX4 expression can alleviate brain damage in VD rats, while knocking out the GPX4 gene exacerbates brain damage (Ming et al., 2023). The reduced activity of GPX4 and disturbances in glutathione metabolism also play crucial roles in ferroptosis (Mou et al., 2019; Tang et al., 2023), further complicating the mechanism of ferroptosis in VCID brain tissues.

**FIGURE 3:**
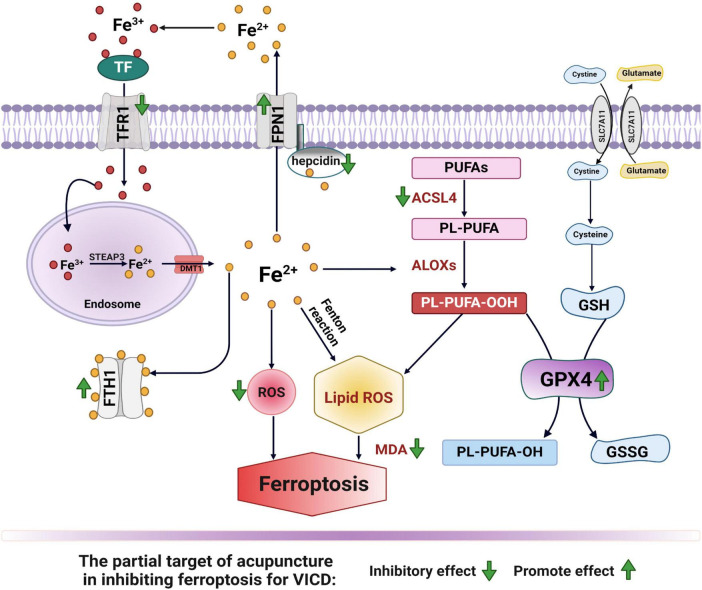
The partial target of acupuncture in inhibiting ferroptosis for VICD. The membrane protein transferrin receptor 1 (TFR1) imports Fe^3+^ into cells after binding to transferrin (TF). STEAP3, a six-transmembrane epithelial antigen of prostate 3, then reduces Fe^3+^ to Fe^2+^ within endosomes. Divalent metal transporter 1 (DMT1) transports Fe^2+^ out of the endosomes. Cytosolic ferritin, composed of ferritin heavy chain 1 (FTH1), stores most of the Fe^2+^, while the membrane protein ferroportin 1 (FPN1) facilitates its export from the cell. Hepcidin internalizes and degrades FPN1, thereby reducing intracellular Fe^2+^ transport. Excess Fe^2+^ can generate reactive oxygen species (ROS) through the Fenton reaction or iron-catalyzed enzyme pathways. Additionally, Fe^2+^ can enhance the activity of lipoxygenases (ALOXs) and accelerate the oxidation of polyunsaturated fatty acids (PUFAs), leading to ferroptosis. The X_<*suprm*>*c*</*suprm*>_ system imports cystine and exports glutamate, with cystine being reduced to cysteine for the production of glutathione (GSH). GSH serves as a substrate for the synthesis of glutathione peroxidase 4 (GPX4), both of which repair cell membrane lipids. GPX4 reduces lipid hydroperoxides (L-OOH) to lipid alcohols (L-OH). Overall, iron overload, an imbalance in the antioxidant system, and the accumulation of lipid peroxides contribute to the ferroptosis observed in VCID. Acupuncture has been shown to exert regulatory effects on these key processes (indicated by green arrows).

Acupuncture can reduce lipid peroxidation by upregulating the expression of antioxidant enzymes such as GPX4 (Wang Q. et al., 2024), SOD (Lin et al., 2024), and CAT in the brain tissue of MCAO/R rat models following stroke. These enzymes effectively scavenge intracellular ROS and reduce lipid peroxidation. GPX4 is an important antioxidant enzyme in ferroptosis, which reduces phospholipid hydroperoxides to protect cell membranes from oxidative damage. By upregulating the expression of GPX4 (Dai et al., 2024; Gao and Yang, 2023), acupuncture significantly lowers lipid peroxidation levels and decreases the expression of MDA in brain tissue of rats with cerebral ischemia or cerebral hemorrhage (Li M.-Y. et al., 2022; Wang et al., 2022; Zhang et al., 2023), potentially inhibiting ferroptosis in VCID (Figure 3).

### 6.3 The regulatory effects of acupuncture on ferroptosis via other metabolic pathways

While lipid metabolism and iron homeostasis are key regulators of ferroptosis, recent studies suggest that glucose metabolism (Zhu K. et al., 2024), amino acid metabolism (Yang et al., 2022a), and neurotransmitter systems (Kim et al., 2021)also play critical roles in ferroptotic cell death. Acupuncture, known for its systemic regulatory effects, may influence ferroptosis not only by modulating lipid peroxidation but also through its impact on glucose utilization, amino acid availability, and glutamate excitotoxicity, thereby reducing oxidative stress and neuronal vulnerability in VCID (Chang et al., 2018).

Glucose metabolism is central to cellular redox homeostasis, as it supplies nicotinamide adenine dinucleotide phosphate, reduced form (NADPH) through the pentose phosphate pathway (PPP), which is essential for regenerating GSH—a key antioxidant that suppresses ferroptosis (Hao et al., 2018). Studies have shown that impaired glucose metabolism exacerbates ferroptotic damage by limiting NADPH availability, thus weakening the cell’s ability to detoxify lipid peroxides. Acupuncture has been reported to enhance glucose uptake and utilization, likely through its effects on AMP-activated protein kinase (AMPK) and insulin signaling pathways (Liu X. et al., 2022). For instance, stimulation at ST36 has been shown to upregulate glucose transporter type 4 (GLUT4) expression, improving glucose transport into neurons and astrocytes in an AD animal model, which may enhance PPP activity and bolster ferroptosis resistance (Ni et al., 2023). Additionally, acupuncture has been found to modulate mitochondrial bioenergetics, optimizing ATP production in the liver damage rat model and have the potential to mitigate ferroptosis-associated metabolic stress (Lee et al., 2022).

Amino acid metabolism, particularly glutamate, cysteine, and glycine, is integral to ferroptosis regulation due to its influence on glutathione synthesis (Poltorack and Dixon, 2022). The cystine-glutamate antiporter system (system Xc^–^) imports cystine, which is converted into cysteine, the rate-limiting precursor for GSH biosynthesis. In ferroptosis, inhibition of system Xc^–^ reduces GSH levels, leading to uncontrolled lipid peroxidation and cell death (Ye et al., 2024). Acupuncture may support cystine uptake and GSH synthesis by modulating Nrf2 signaling, a key pathway regulating antioxidant responses. Electroacupuncture (EA) at DU20 and Shenshu (BL23) has been associated with increased Nrf2 activation and enhanced cysteine availability in ischemic stroke rat model, potentially contributing to greater resistance against ferroptotic stress (Yang et al., 2025). Furthermore, acupuncture may regulate the metabolism of methionine and glycine, both of which are critical for glutathione synthesis, further reinforcing cellular antioxidant defenses (Li H. et al., 2024).

Glutamate excitotoxicity is a major driver of oxidative stress and neuronal damage in neurodegenerative diseases, including VCID. Excessive extracellular glutamate inhibits system Xc^–^, reducing cystine uptake and depleting GSH, thereby promoting ferroptosis. This mechanism is particularly relevant in ischemic and hypoxic conditions, where glutamate accumulation exacerbates neuronal injury. A review article discusses how acupuncture can potentially modulate glutamate receptors and excitatory amino acid transporters (EAATs), suggesting that acupuncture treatment effects may be underpinned by its intervention in the dysregulated glutamate system (Tu et al., 2019). While specific studies directly linking acupuncture to increased EAAT activity and reduced ferroptosis-induced neurotoxicity are limited, the general modulation of glutamatergic neurotransmission by acupuncture indicates a potential for such effects. By enhancing EAAT activity, acupuncture may reduce extracellular glutamate levels, thereby alleviating excitotoxicity and potentially mitigating ferroptosis-related neuronal damage.

### 6.4 Acupuncture effects in comparison with pharmacological inhibitors and dietary interventions for ferroptosis regulation

Pharmacological agents like liproxstatin-1 are specific inhibitors of lipid peroxidation, functioning by inhibiting GPX4 activity and preventing the accumulation of lipid hydroperoxides, which are key mediators of ferroptotic cell death (Fan et al., 2020). While effective in ferroptosis-sensitive cell lines, liproxstatin-1 and similar compounds can have narrow therapeutic windows, as they do not address the broader metabolic disruptions that contribute to ferroptosis. Long-term use of such pharmacological agents could potentially disrupt essential cellular functions, such as lipid metabolism and antioxidant defense (Zilka et al., 2017). In contrast, acupuncture modulates ferroptosis through a more holistic mechanism, influencing multiple pathways related to glutathione metabolism, lipid homeostasis, and iron regulation. By stimulating specific acupoints such as DU20 and ST36 in rats model of MCAO/R, acupuncture can increase antioxidant enzyme activity (GPX4, SOD, CAT), reduce lipid peroxidation, and optimize iron metabolism—all of which contribute to enhanced neuroprotection and decreased ferroptotic damage (Yang et al., 2025). Additionally, acupuncture has been shown to reduce neuroinflammation and improve cerebral blood flow, addressing underlying metabolic and vascular issues that contribute to VCID (Li et al., 2023; Wang et al., n.d.).

Dietary interventions, particularly the intake of omega-3 PUFAs, have been shown to modulate lipid metabolism and reduce lipid peroxidation, making them potential adjuncts to ferroptosis inhibition (Mortensen et al., 2023). Omega-3 PUFAs, particularly eicosapentaenoic acid (EPA) and docosahexaenoic acid (DHA), have anti-inflammatory and antioxidant properties that reduce oxidative stress and protect against ferroptosis by incorporating into cell membranes, stabilizing phospholipids, and reducing their vulnerability to peroxidation (Suda et al., 2024). However, the effectiveness of omega-3 PUFAs in ferroptosis inhibition is largely dependent on the dietary intake levels and bioavailability of these fatty acids. Acupuncture can complement dietary interventions by enhancing the absorption of omega-3 PUFAs and promoting their incorporation into cellular membranes. For example, acupuncture at ST36 has been shown to stimulate intestinal absorption and optimize lipid metabolism in metabolic syndrome secondary mouse model, which may enhance the bioavailability and bioactivity of omega-3 PUFAs (Han et al., 2020). Additionally, acupuncture’s ability to regulate metabolic pathways could enhance the protective effects of omega-3 PUFAs, particularly in the context of ferroptosis-associated neurodegeneration.

The combination of acupuncture with pharmacological inhibitors or dietary interventions holds great potential for synergistic ferroptosis inhibition in VCID treatment. For example, acupuncture may optimize the bioavailability and efficacy of liproxstatin-1 by improving circulation and cellular absorption, while also mitigating its potential side effects by supporting metabolic balance and tissue regeneration. Similarly, omega-3 PUFA supplementation, when combined with acupuncture, could enhance the incorporation of these protective lipids into membranes, thereby further reducing lipid peroxidation and promoting membrane integrity during ferroptosis.

### 6.5 Selective regulation of ferroptosis by acupuncture: distinction from apoptosis and necroptosis

Acupuncture’s role in ferroptosis regulation is highly specific, primarily targeting iron homeostasis and lipid peroxidation, which are hallmark mechanisms of ferroptotic cell death. Unlike apoptosis, which is caspase-dependent, and necroptosis, which is mediated by droped in adenosine triphosphate (ATP) levels, ferroptosis is driven by iron overload and the accumulation of lipid peroxides (Yang et al., 2022b; Table 1).

**TABLE 1:** Comparison of morphological, biochemistry and genetics features of apoptosis and necrosis.

Cell death type	Morphological features	Biochemical features	Core genes	Inducers	Inhibitors
Ferroptosis	Mitochondrial shrinkage and morphological abnormalities (increased membrane density, diminished or vanished cristae, ruptured outer membrane), with normal nucleus	Iron accumulation, lipid peroxidation, inhibition of SLC7A11/GSH/GPX4	GPX4, TfR1, FTH1, SLC7A1, NRf1, NCOA4, PRDX3, ALOXs, ACSL4	Erastin, RSL3	Ferrostatin-1, liproxstatin-1, vitamin E, desferoxamine
Apoptosis	Cell shrinkage and convolution. Pyknosis and karyorrhexis. Intact cell membrane. Cytoplasm retained in apoptotic bodies.	DNA fragmentation, activation of caspase pathway, no inflammation	Caspase, Bcl-2, Bax, P53, Fas	FASL, DCC, UNC5B	zVAD-FMK, XIAP, c-IAP1
Necroptosis	Cell swelling. Karyolysis, pyknosis, and karyorrhexis. Disrupted cell membrane. Cytoplasm released.	Drop in ATP levels, inflammation usually present	RIP1, RIP3, LEF1	zVAD-fmk, TNF-α	Necrostatin-1, NSA

GPX4, glutathione peroxidase 4; TfR1, transferrin receptor 1; FTH1, ferritin heavy chain 1; SLC7A1, solute carrier family 7 member 1; NRF1, nuclear respiratory factor 1; NCOA4, nuclear receptor coactivator 4; PRDX3, peroxiredoxin 3; ALOXs, lipoxygenases; ACSL4, Acyl-CoA synthetase long-chain family member; RSL3, Ras selective lethal 3; Caspase, cysteine aspartate-specific protease; Bcl-2, B-cell lymphoma 2; Bax, Bcl-2-associated X protein; P53, tumor protein P53; Fas, fas cell surface death receptor; FASL, fas ligand; DCC, deleted in colorectal carcinoma; UNC5B, Unc-5 netrin receptor B; zVAD-FMK, pan-caspase inhibitor; XIAP, X-linked inhibitor of apoptosis protein; c-IAP1, cellular inhibitor of apoptosis protein 1; RIP1, receptor-interacting protein kinase 1; RIP3, receptor-interacting protein kinase 3; LEF1, lymphoid enhancer-binding factor 1; TNF-α, tumor necrosis factor alpha; NSA, necrosulfonamide.

Existing studies indicate that acupuncture exerts its effects on ferroptosis by modulating key regulators such as GPX4, ACSL4, and TfR1 (Wang G.-L. et al., 2023), which are not directly involved in apoptosis or necroptosis. For example, acupuncture has been shown to upregulate GPX4 activity in MCAO/R rats model, which mitigates lipid peroxidation and protects neuronal membranes from ferroptosis-induced damage. Additionally, acupuncture also inhibits the expression of ACSL4 in MCAO/R rats model, an enzyme critical for incorporating PUFAs into membrane phospholipids, thereby reducing susceptibility to ferroptosis.

In contrast, apoptosis is characterized by the activation of caspase-3, caspase-7, and caspase-9, leading to DNA fragmentation and cell shrinkage (Elmore, 2007). Acupuncture studies have demonstrated its potential to regulate apoptosis via pathways such as the Bcl-2/Bax axis or the PI3K/Akt pathway (Yang et al., 2023), yet these mechanisms do not overlap significantly with its ferroptosis-regulating effects. Similarly, necroptosis involves the RIPK1-RIPK3-MLKL cascade (Dhuriya and Sharma, 2018), which is not significantly altered by acupuncture in ferroptosis-related studies. Thus, while acupuncture may have regulatory effects on multiple cell death pathways, its ability to selectively influence ferroptosis appears to be mediated by its impact on iron metabolism and oxidative stress, rather than by direct modulation of apoptotic or necroptotic machinery.

### 6.6 Specific acupoints for ferroptosis regulation and their neurovascular relevance

The regulation of ferroptosis by acupuncture is closely associated with its ability to modulate iron homeostasis, oxidative stress, and lipid metabolism. While acupuncture is known for its systemic effects, certain acupoints have demonstrated greater efficacy in ferroptosis regulation, particularly Baihui (DU20), Zusanli (ST36), and Shuigou (DU26). These points are functionally relevant to the brain vascular network, playing crucial roles in enhancing cerebral circulation, stabilizing the BBB, and modulating metabolic pathways that influence ferroptosis susceptibility.

DU20, located at the vertex of the skull, is directly linked to cerebral perfusion (Lin et al., 2016) and neurovascular protection (Liu et al., 2018). Research suggests that its stimulation improves regional cerebral blood flow (rCBF) and helps maintain BBB integrity in rat intracerebral hemorrhage models via the RhoA/ROCK II/MLC 2 signaling pathway, preventing excessive iron influx into neuronal tissues (Zhang C. et al., 2024). Additionally, Baihui acupuncture modulates key ferroptosis regulators such as TfR1, FPN1, and all of which are involved in iron transport, lipid peroxidation control, and antioxidant defense. ST36, a major acupoint on the lower leg, has been widely studied for its effects on systemic metabolism and oxidative stress regulation (Jiang et al., 2023). It has been shown to enhance antioxidant enzyme activity, including GPX4, SOD, and CAT, reducing oxidative damage and mitigating ferroptosis in MCAO/R Rats (Zhu W. et al., 2024). Additionally, ST36 influences lipid metabolism and glucose homeostasis (Lee et al., 2011) in a rat model of insulin-dependent diabetes, both of which are critical factors in neuronal resilience to ferroptotic damage. DU26, located at the midline of the philtrum, has been traditionally used for its neuroprotective effects, particularly in ischemic stroke and cerebral hypoxia. Acupuncture at DU26 is believed to promote cerebrovascular autoregulation, reduce iron accumulation in brain tissues, and protect against lipid peroxidation-induced neuronal injury in cerebral hemorrhage rat model (Liu et al., 2018). By stabilizing iron metabolism and preserving membrane phospholipid integrity, DU26 acupuncture may help limit ferroptosis-related neurodegeneration (Table 2).

**TABLE 2:** Neurovascular mechanisms and ferroptosis-regulating pathways of key acupoints (DU20, ST36, DU26).

Acupoint code and name	Meridian	Typical location	Neurovascular relevance	Model	Improvement of neurological function	Important molecular alterations	References
DU20 (Baihui)	Governor Vessel (Du Meridian)	At the vertex of the head on the midline, in the intersection of a line connecting the apexes of the ears.	Enhances cerebral blood flow and stabilizes the superior sagittal sinus circulation, improving neurovascular autoregulation.	MCAO	NFS (Bederson)	FTH1↑,GPX4↑, and ACSL4↓	Wang G.-L. et al., 2023
				ICH	NFS (Longa mNSS)	TfR1↓	Chen et al., 2022
				MCAO/R	NFS (Longa)	GSH↑, and MDA↓	Wu et al., 2023
				ICH	NFS (Ludmila Belayev)	FTH1↑,GPX4↑, and MDA↓	Li M.-Y. et al., 2022
				ICH	NFS (Ludmila Belayev)	GPX4↑, GSH↑,and MDA↓	Dai et al., 2024
				MCAO/R	NFS (Longa)	ROS↓and MDA↓	Yang, 2024
				MCAO/R	NFS (Garcia)	ROS↓and MDA↓	Wang et al., 2022
				MCAO/R	NFS (Longa mNSS)	GPX4↑, GSH↑,and ROS↓	Liang, 2023
				ICH	NFS (Ludmila Belayev)	MDA↓	Li M. et al., 2021
				4 - VO	NFS (Longa)	SOD↑	Zuo et al., 2017
				MCAO/R	–	ROS↓, MDA↓,and SOD↑	Lin et al., 2024
				MCAO/R	NFS (Longa)	MDA↓, GSH↑, and SOD↑	Lin et al., 2015
				MCAO/R	NFS (Garcia)	BDNF↑	Kim et al., 2012
ST36 (Zusanli)	Stomach Meridian	On the lower leg, approximately 3 cun below the lateral knee, 1 finger-breadth lateral to the anterior crest of the tibia.	Improves systemic microcirculation by regulating peripheral vascular resistance and endothelial function.	MCAO/R	NFS (Longa mNSS)	NSF (Longa mNSS)	Liang, 2023
				MID	–	SOD↑	Zhang et al., 2014
				4 - VO	NFS (Longa)	SOD↑	Zuo et al., 2017
				MID	–	SOD↑	Liu et al., 2006
				MCAO/R	NFS (Longa)	BDNF↑	Tao et al., 2016
				MCAO/R	NFS (Longa)	BDNF↑	Chen et al., 2012
DU26 (Shuigou)	Governor Vessel (Du Meridian)	At the junction of the upper and middle thirds of the philtrum on the midline of the upper lip.	Activates the trigeminal-cerebral reflex to increase cortical blood flow and prevent hypoxia-induced damage.	PSD	NFS (Longa)	BDNF↑and SOD↑	Sun et al., 2022
				MCAO/R	NFS (Garcia)	ROS↓and MDA↓	Wang et al., 2022
				ICH	NFS (Zausinger)	GPX4↑, GSH↑,and MDA↓	Gao and Yang, 2023
				MCAO/R	NFS (Zausinger)	GPX4↑, GSH↑, TfR1↓and MDA↓	Wang Q. et al., 2024
				MCAO/R	NFS (Bederson)	FTH1↑and MDA↓	Zhang X. et al., 2024
				MCAO	NFS (Bederson)	FTH1↑,GPX4↑, and ACSL4↓	Wang G.-L. et al., 2023

MCAO, middle cerebral artery occlusion; MCAO/R, middle cerebral artery occlusion/reperfusion); ICH, intracerebral hemorrhage; 4-VO, four-vessel occlusion; MID, multi-infarct dementia; PSD, post-stroke depression; NFS, neurological function score; FTH1, ferritin heavy chain 1; FTH1, ferritin heavy chain 1; TfR1, transferrin receptor 1; ROS, reactive oxygen species; MDA, malondialdehyde; SOD, superoxide dismutase; GSH, glutathione; GPX4, glutathione peroxidase 4; BDNF, brain-derived neurotrophic factor; ACSL4, Acyl-CoA synthetase long-chain family member 4.

These acupoints are functionally integrated into the brain vascular network, influencing cerebral circulation, oxidative stress regulation, and lipid peroxidation pathways. Their ability to improve blood flow and strengthen BBB function may reduce neuronal exposure to iron overload and ROS, thereby decreasing ferroptotic vulnerability. The targeted use of these acupuncture points offers a multi-faceted approach to ferroptosis regulation, complementing pharmacological strategies for treating VCID. Further research is needed to explore the precise mechanisms by which these acupoints exert their effects and to determine their potential in combination therapies aimed at ferroptosis inhibition (Table 2).

### 6.7 Long-term effects of acupuncture on ferroptosis susceptibility

Evidence indicates that repeated acupuncture stimulation yields sustained neuroprotective benefits by stabilizing iron homeostasis and enhancing antioxidant responses (Huo et al., 2014). Chronic or repeated interventions—such as regular EA at acupoints like DU20 and ST36 of stroke models—have been shown to persistently upregulate protective enzymes, including GPX4, while maintaining balanced expression of key iron transporters like TfR1 and FTH1 (Table 3). This cumulative effect helps to mitigate excessive iron accumulation and reduces the formation of lipid peroxides, thereby decreasing the cellular susceptibility to ferroptosis over time. In addition, repeated acupuncture appears to exert anti-inflammatory effects and promote overall metabolic homeostasis (Shen et al., n.d.). By modulating pathways related to glucose and amino acid metabolism, acupuncture may reinforce the cell’s intrinsic antioxidant defenses and improve mitochondrial function, which in turn supports both peroxisomal activity and redox balance (Chen H. et al., 2023). This integrated modulation not only helps to counteract acute oxidative stress but also establishes a long-term cellular environment that is more resistant to the deleterious cascades leading to ferroptosis, ultimately contributing to enhanced neuronal resilience (Table 3).

**TABLE 3:** Acupuncture intervention protocols and long-term effects on ferroptosis regulation.

Type	Frequency	Intervention intensity	Intervention time	Detection time	Acupoints	Model	Long-term effects	Molecular alterations	References
EA	3.85/6.25 Hz	0.8∼1.3 mA	EA 30 min	1 d, 2 d, 3 d	DU26, DU20	MCAO	Antioxidant and mitochondrial protection.	FTH1↑GPX4↑ACSL4↓	Wang G.-L. et al., 2023
EA	2/15 Hz	2 mA	EA 30 min	3 d, 7 d, 14 d	DU20, DU14	ICH	Improve iron metabolism.	TfR1↓	Chen et al., 2022
EA	2/15 Hz	1–2 mA	EA 20 min	7 d	DU20, DU16, DU14	MCAO/R	Neuroprotective effect.	GSH↑MDA↓	Wu et al., 2023
SA	180 ± 20 r/min	–	SA 30 min	6 h, 3 d, 7 d	DU20, GB7	ICH	Mitochondrial protective effect.	FTH1↑GPX4↑MDA↓	Li M.-Y. et al., 2022
MA	–	–	MA 30 min	1 d	PC6, DU26	MCAO/R	Improve iron metabolism.	FTH1↑MDA↓	Zhang X. et al., 2024
EA	2/15 Hz	1 mA	EA 30 min	1 d, 3 d	DU26, SP6, PC6	MCAO/R	Regulating iron metabolism and enhancing antioxidant capacity.	GPX4↑ GSH↑ TfR1↓ MDA↓	Wang Q. et al., 2024
MA	–	–	MA 30 min	1 d, 3 d, 7 d	GB7	ICH	Enhancing brain antioxidant capacity.	GPX4↑ GSH↑ MDA↓	Dai et al., 2024
MA	–	–	MA 30 min	3 d, 7 d, 14 d	DU20	MCAO/R	Regulating lipid peroxidation.	ROS↓ MDA↓ ACSL4↓	Yang, 2024
EA	2/15 Hz	2 mA	–	3 d	DU26, SP6, PC6	ICH	Enhancing brain antioxidant capacity.	MDA↓ GSH↑ GPX4↑	Gao and Yang, 2023
MA	–	–	MA 30 min		DU20, DU26, DU14	MCAO/R	Improve iron metabolism.	ROS↓ MDA↓	Wang et al., 2022
EA	2/15 Hz	1 mA	–	1 d, 3 d, 7 d	DU20, ST6	MCAO/R	Improve iron metabolism.	GPX4↑ GSH↑ ROS↓	Liang, 2023
MA	–	–	MA 30 min	1 d, 3 d, 7 d	DU20, GB7	ICH	Neuronal and mitochondrial protection.	MDA↓	Li M. et al., 2021
EA	40/50 Hz	–	EA 20 min	1 d, 3 d	DU4, DU20, ST36	4 - VO	Reduce mitochondrial damage.	SOD↑	Zuo et al., 2017
MA	–	–	MA 30 min	7 d	CV17, CV12, CV6, SP10, ST36	MID	Improve cerebral blood flow and protect mitochondria.	SOD↑	Zhang et al., 2014
EA	2/20 Hz	–	EA 30 min	7 d	DU20, DU24	MCAO/R	Reduce oxidative stress.	ROS↓ MDA↓ SOD↑	Lin et al., 2024
EA	5/20 Hz	1–3 mA	EA 30 min	7 d	DU20, DU24	MCAO/R	Reduce oxidative stress.	MDA↓ GSH↑ SOD↑	Lin et al., 2015
EA	1/20 Hz	1 mA	EA 30 min	3 d	LI11, ST36	MCAO/R	Neuroprotective effect.	BDNF↑	Tao et al., 2016

### 6.8 Acupuncture-targeting ferroptosis in VCID: A double-edged sword

Acupuncture presents a multi-pathway therapeutic approach for ferroptosis suppression in VCID, distinguishing itself from pharmacological inhibitors that target only specific molecular mechanisms. By modulating iron homeostasis, acupuncture reduces iron overload through the regulation of FPN1, TfR1, and hepcidin, thereby preventing excessive iron accumulation in ferroptosis-prone brain regions (Wang Q. et al., 2024). Additionally, acupuncture influences lipid peroxidation pathways by downregulating ACSL4 and upregulating GPX4, which helps stabilize neuronal membranes and mitigate oxidative lipid damage (Dai et al., 2024). Beyond its effects on ferroptosis regulation, acupuncture enhances antioxidant defense mechanisms by activating Nrf2 signaling, increasing GSH levels, and upregulating SOD and CAT, counteracting ferroptotic oxidative stress (Lin et al., 2024). Unlike iron chelation therapy or synthetic ferroptosis inhibitors, acupuncture is non-invasive and associated with fewer systemic side effects, reducing the risk of metabolic disruptions. Furthermore, given that chronic cerebral hypoperfusion exacerbates ferroptosis susceptibility in VCID, acupuncture at DU20 and ST36 has been reported to enhance cerebral blood flow, alleviate ischemia-induced oxidative stress, and promote neurovascular repair, indirectly suppressing ferroptotic damage (Gao and Yang, 2023; Wang G.-L. et al., 2023). Its adaptability for precision medicine approaches further supports its potential as a personalized intervention, allowing treatments to be tailored based on individual metabolic and neurovascular status.

The efficacy of acupuncture in ferroptosis modulation for VCID may vary significantly among individuals due to genetic differences, metabolic profiles, and vascular health, making treatment outcomes difficult to predict. Additionally, the lack of standardization in acupoint selection, stimulation techniques, and treatment duration may contribute to inconsistent therapeutic effects across studies, limiting its reproducibility in clinical research. Unlike pharmacological approaches with precise dosing, acupuncture outcomes are highly dependent on practitioner expertise, which poses challenges for scalability and standard clinical implementation. Addressing these issues through standardized protocols and evidence-based optimization of acupuncture parameters will be essential for ensuring reliable and reproducible results in ferroptosis-targeting VCID therapy.

## 7 Membrane phospholipid composition dictates ferroptosis susceptibility in VICD neurons

**FIGURE 4:**
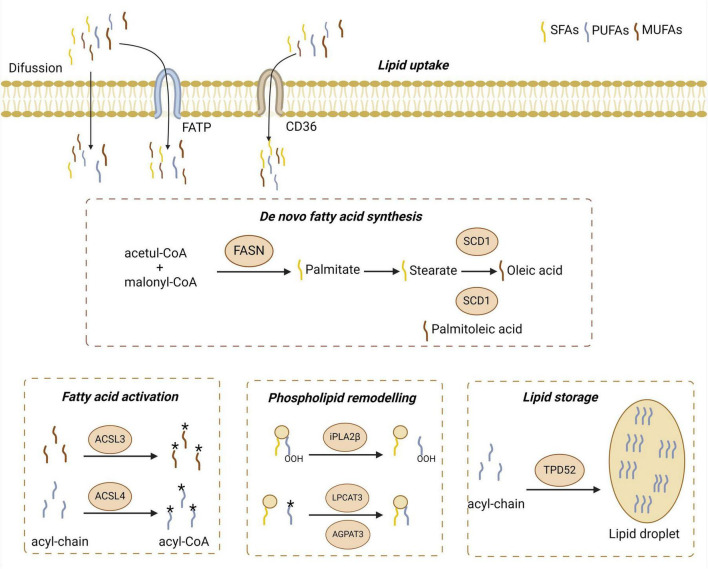
The uptake, activation, remodeling, and storage pathways of lipid metabolism in ferroptosis. Free fatty acids enter cells via diffusion or transporters like CD36 and FATPs. Monounsaturated fatty acids (MUFAs) can be synthesized *de novo*, starting from malonyl-CoA and acetyl-CoA, elongated and desaturated by SCD1, and activated by ACSL3, which incorporates them into phospholipids to inhibit lipid peroxidation. In contrast, polyunsaturated fatty acids (PUFAs) are taken up from the extracellular environment and activated by ACSL4. Phospholipid remodeling enzymes such as PLA2G6, LPCAT3, and AGPAT3 regulate PUFA integration, influencing membrane oxidizability. TPD52, a lipid droplet synthesis enzyme, may protect against ferroptosis by sequestering PUFAs into lipid droplets, preventing their incorporation into membrane phospholipids. FASN, fatty acid synthase; SFA, saturated fatty acid; FATPs, fatty acid transport proteins; SCD1, Stearoyl-CoA desaturase 1; ACSL3, Acyl-CoA synthetase long-chain family member 3; ACSL4, Acyl-CoA synthetase long-chain family member 4; PLA2G6, phospholipase A2 group VI; LPCAT3, lysophosphatidylcholine acyltransferase 3; AGPAT3, 1-acylglycerol-3-phosphate O-acyltransferase 3; TPD52, tumor protein D52. The asterisk “*” denotes an esterification reaction. Specifically, it highlights key enzymatic steps—such as the activation of unsaturated fatty acids into acyl-CoA forms and the reassembly of PUFAs into membrane phospholipids—where enzymatic catalysis, energy input, or molecular modifications are typically required.

Ferroptosis is closely related to intracellular lipid metabolism homeostasis, with both lipid peroxidation and antioxidation—two key systems of ferroptosis—being highly regulated by lipid metabolism (Jiang et al., 2021). Lipids, especially phospholipids, are crucial for maintaining the structure and function of cell membranes (Zou et al., 2020a). Lipid metabolism influences the types and amounts of fatty acids bound to membrane phospholipids (mPL), thereby affecting the composition of membrane phospholipids and the interactions between lipid molecules (Qiu et al., 2024). This, in turn, alters the flux of various substances and signal transduction across the membrane, ultimately impacting cellular activity and function. The uptake and metabolism of polyunsaturated fatty acids (PUFAs), and the synthesis of PUFA phospholipids (PUFA-PLs), crucially shape cellular sensitivity to ferroptosis. Any metabolite, protein, or process that alters these processes seems likely to impact ferroptosis sensitivity (Pope and Dixon, 2023; Figure 4).

In the glycerol backbone of membrane phospholipids, each glycerol molecule can bind two fatty acids. PUFAs are typically esterified to glycerol molecules. A lot of PUFAs contain multiple bis-allylic bonds, which are highly susceptible to oxidation and serve as primary sites for lipid peroxidation during ferroptosis (Zou et al., 2020a). In contrast, monounsaturated fatty acids (MUFAs) lack bis-allylic bonds and can inhibit membrane lipid peroxidation and ferroptosis by replacing PUFAs (Perez et al., 2020).

Studies have shown that the binding and peroxidation of PUFAs with membrane phospholipids are prerequisites for ferroptosis (Jiang et al., 2021). This process can be summarized as follows: free PUFAs are first activated by ACSL4 and form PUFA-CoA with CoA (Doll et al., 2017). Subsequently, under the catalysis of LPCAT3 (lysophosphatidylcholine acyltransferase 3), PUFA-CoA is esterified and incorporated into membrane phospholipids to form PUFA-mPL. PUFA-mPL is then oxidized by ROS to form phospholipid hydroperoxides or converted into phospholipid hydroperoxides through the catalysis of enzymes such as ALOXs (Kuhn et al., 2015) or cytochrome P450 oxidoreductase (POR) (Zou et al., 2020b). Research indicates that ACSL4 and LPCAT3 (Jiang et al., 2021) are involved in the synthesis of PUFA-mPL, and both have been identified as critical drivers of ferroptosis. Their inactivation or downregulation significantly increases cellular resistance to ferroptosis. ALOXs and POR promote the oxidation of PUFA-mPL, and inhibiting their activity renders cells less sensitive to ferroptosis.

Similar phenomena are observed in brain tissue. For instance, upregulation of ACSL4 is associated with neuronal death and ischemia-reperfusion injury post-ischemia. A study demonstrated that in a focal cerebral ischemia mice model, ACSL4 promotes neuronal death by facilitating neuronal ferroptosis (Cui Y. et al., 2021). Knocking out ACSL4 alleviated ischemic brain injury, whereas overexpression of ACSL4 exacerbated cerebral ischemia. A clinical study indicated that the overexpression of ACSL4 may be regulated by miR-347, which is found to be elevated in patients with ischemic stroke, subsequently leading to the upregulation of ACSL4 expression.

ALOXs are central players in ferroptosis (Qin et al., 2023). Activation of ALOXs initiates the pool of lipid hydroperoxides crucial for ferroptosis, accelerating lipid autoxidation and driving process of the human embryonic kidney cell death (Shah et al., 2018). Also, ALOXs are highly expressed after cerebral ischemia, and their inhibitors can suppress ferroptosis and alleviate brain damage. For instance, compared to the sham group, ALOX expression in MCAO rats was elevated between 6 and 72 h. However, after treatment with Buyang Huanwu Decoction, ALOX expression significantly decreased at 24, 48, and 72 h. Additionally, inflammation-induced injury was reduced, and neurological function was improved following ischemia-reperfusion in rats (Gao, 2023). Another study also suggests (Karatas et al., 2018) that ALOX plays an important role in brain injury following MCAO. Contrary to the brain-damaging effects of increased ALOX activity during MCAO, systemic administration of the ALOX inhibitor LOX Block-1 (LB1) 2 h after permanent focal cerebral ischemia significantly reduced infarct size at 24 h post-ischemia. The treatment group also showed improvements in behavioral and health parameters.

**FIGURE 5:**
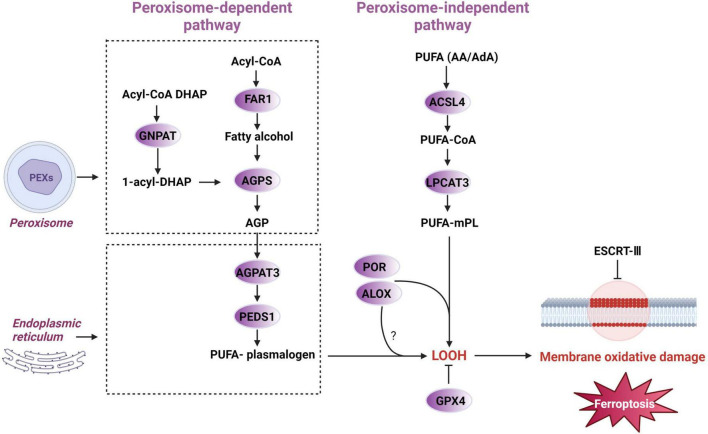
Peroxisome-dependent and independent initiation of ferroptotic pathways. Polyunsaturated fatty acid (PUFA), arachidonic acid/adrenic acid (AA/AdA), acyl-CoA synthetase long-chain family member 4 (ACSL4), polyunsaturated fatty acid -coenzyme A (PUFA-CoA), lysophosphatidylcholine acyltransferase 3 (LPCAT3), polyunsaturated fatty acid- membrane phospholipid (PUFA-mPL), lipoxygenase (ALOX), cytochrome P450 oxidoreductase (POR), lipid hydroperoxides (LOOH), glutathione peroxidase 4 (GPX4), endosomal sorting complexes required for transport-III (ESCRT-III), alkylglycerone phosphate synthase (AGPS), 1-O-alkyl-glycerol-3-phosphate (AGP), 1-acylglycerol3-phosphate O-acyltransferase 3 (AGPAT3), dihydroxyacetone phosphate (DHAP), fatty acyl-CoA reductase 1 (FAR1), glyceronephosphate O-acyltransferase (GNPAT), plasmanylethanolamine desaturase 1 (PEDS1).

Additionally, in the ischemic mouse brains, membrane phospholipids are selectively degraded, leading to an increased release of PUFAs, predominantly arachidonic acid (AA) (Drgová et al., 2004). Arachidonic acid/adrenic acid (AA/AdA), which contain three bis-allylic bonds, are primary substrates for lipid peroxidation in ferroptosis (Wang Y. et al., 2023). This suggests that the types and amounts of PUFAs in membrane phospholipids are crucial determinants of cell survival under ferroptotic conditions (Yan N. et al., 2021). Conversely, lipid peroxidation repair enzymes like GPX4 and the endosomal sorting complexes required for transport (ESCRT)-III membrane repair machinery can prevent ferroptosis (Tang and Kroemer, 2020; Figure 5, the peroxisome- independent pathway).

**FIGURE 6:**
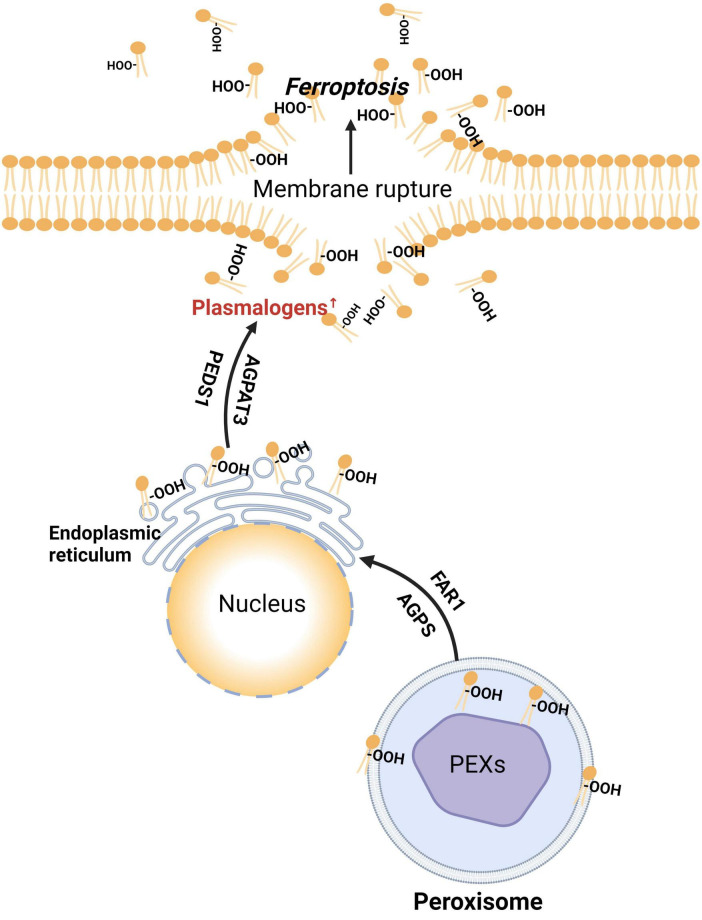
The pathway of plasmalogens and peroxisomes in ferroptosis induction. PEXs, peroxisome biogenesis factors; AGPS, alkylglycerone phosphate synthase; FAR1, fatty acyl-CoA reductase 1; AGPAT3, 1-acylglycerol-3-phosphate O-acyltransferase 3; -OOH, lipid hydroperoxides; PEDS1, plasmalogen ether desaturase 1.

In summary, dysregulated lipid metabolism may be the pathological basis for neuronal ferroptosis. Abnormal lipid metabolism alters the types and amounts of PUFAs bound to membrane phospholipids, thereby affecting neuronal sensitivity to ferroptosis. This is likely a significant factor contributing to cognitive impairment in VCID (Figure 6).

### 7.1 The regulatory effects of acupuncture on PUFA-mPL synthase

Multiple studies have shown that acupuncture may regulate VCID by modulating ACSL4 (Wang G.-L. et al., 2023; Yang, 2024; Zhang et al., 2023). For example, research indicates that EA at DU26 and DU20 significantly reduces ACSL4 expression in the brain tissue of stroke model MCAO/R rats, thereby inhibiting ferroptosis to alleviate I/R injury (Wang G.-L. et al., 2023). This effect could be attributed to EA’ s ability to lower ACSL4 expression in brain tissue during ischemia and hypoxia, thereby reducing the catalysis of PUFA conjugation with coenzyme A and decreasing the incorporation of these fatty acids into phospholipids, particularly PUFA-enriched phospholipids.

Furthermore, acupuncture can regulate LPCAT3 expression, which affects PUFA esterification and inhibits ferroptosis. According to studies, the “Shugan Tiaoshen” acupuncture treatment effectively reduces the proteins and mRNA expression levels of LPCAT3 and ACSL4 in the brain tissue of ischemic stroke rats, resulting in neuroprotective effects (Wu R. et al., 2024). In addition, EA at the Shenmen (HT7) and Tongli (HT5) acupoints of the heart meridian can decrease the levels of ACSL4 and LPCAT3 proteins and mRNA, hence offering protection against myocardial ischemia (Wang, 2023). Moreover, acupuncture can influence ALOXs. However, this mechanism has only been identified in acupuncture therapies for asthma (Tang et al., 2023) and neuropathic pain (Wan et al., 2023), and it has not been observed in VCID-associated disorders. This could be an appealing target for future study into how acupuncture inhibits ferroptosis in the treatment of VCID.

### 7.2 The regulatory effects of acupuncture on membrane phospholipid composition

Acupuncture has been found to be effective in regulating phospholipid metabolism and membrane phospholipid composition. A study on VCID patients revealed alterations in plasma metabolomics profiles before and after a 3-month course of acupuncture treatment, showing notable disparities in the metabolomics profiles of all individuals. Following therapy, the expression levels of phosphatidylcholine (PC), lysophosphatidylcholine (LPC), sphingomyelin (SM), and sphinganine (C16) were substantially increased compared to the levels observed before treatment. DL-carnitine, L-carnitine, SM [d18:0/16:1(9Z)], and SM (d18:0/22:0) were all considerably reduced following treatment, with statistical significance (*P* < 0.05) (Ma, 2023). The finding indicates that acupuncture can potentially affect the plasma metabolomics profile of VCID patients, either by directly regulating certain processes or by enhancing the absorption and metabolism of medications in the body. Moreover, EA can promote skin wound healing in full-thickness skin defect rat models by improving lipid metabolism and inhibiting ferroptosis in local tissues (Du et al., 2023). The results showed that blood flow perfusion and wound healing in the EA group improved more rapidly than in the model group. GPX4 and GSH levels, associated with ferroptosis, were higher in the EA group, while ACSL4 and MDA levels were lower. Additionally, lipid metabolomics analysis in this study revealed that EA regulated 37 lipid metabolites, including phospholipids, lysophospholipids, triglycerides, acylcarnitines, sphingolipids, and fatty acids, all key components of the cell membrane.

Additionally, studies have shown that acupuncture regulates phosphatidylinositol (PI) levels. PI plays a crucial role in cellular signal transduction and membrane dynamics. Acupuncture may improve PI synthesis and metabolism by increasing PI synthase expression, which strengthens the cell’s resistance to ferroptosis. Studies have demonstrated that EA at GV26 can effectively reduce the overexpression of PI system-related factors such as inositol 1,4,5-trisphosphate (IP3), diacylglycerol (DAG), and calmodulin (CaM) in acute cerebral ischemia rats, alleviating cerebrovascular spasms (Li J. et al., 2022). It is important to emphasize that the studies listed above mostly concentrate on peripheral blood, and there is a scarcity of comprehensive study on the composition of phospholipids in the cell membranes of the central nervous system in VCID. Hence, this might potentially serve as a substantial focal point for acupuncture research.

## 8 Peroxisomes and their synthesized plasmalogens heighten ferroptosis susceptibility

Peroxisomes are organelles closely related to lipid metabolism, with functions including the promotion of fatty acid β-oxidation, the removal of free radicals, and the synthesis of plasmalogens, which are ether phospholipids (ePLs) (He et al., 2023; Ying et al., 2023). Recent studies have demonstrated that peroxisomes independently participate in the regulation of ferroptosis, being the only organelles capable of generating plasmalogens (Cui W. et al., 2021; Tang and Kroemer, 2020). The plasmalogens produced by peroxisomes consist of two PUFAs linked to a glycerol molecule via ether and ester bonds, respectively. Since ether bonds are more susceptible to oxidation than ester bonds, cells with a higher content of ePLs are more prone to ferroptosis (Cedillo et al., 2023; Qiu et al., 2024; Zou et al., 2020a). Research has shown that AGPS (alkyl-dihydroxyacetone phosphate synthase) and FAR1 (fatty acid reductase 1) are key enzymes in the biosynthesis of ePLs, and enhancing their expression can promote neuronal ferroptosis (Zou et al., 2020a). The precursor 1-O-alkyl-glycerol-3-phosphate (AGP) is synthesized by FAR1 and AGPS and then transported to the endoplasmic reticulum (ER) for further biosynthetic reactions involving 1-acylglycerol-3phosphate O-acyltransferase 3 (encoded by AGPAT3). Finally, the ER-resident enzyme plasmanylethanolamine desaturase 1 (PEDS1, also known as TMEM189) mediates the production of PUFA plasmalogen. Importantly, the provision of liposomal nanoparticles composed of purified plasmalogens to cells was sufficient to confer sensitivity to ferroptosis by lipid peroxidation, further confirming the implication of plasmalogens in ferroptosis (Tang and Kroemer, 2020; Figure 6).

**FIGURE 7:**
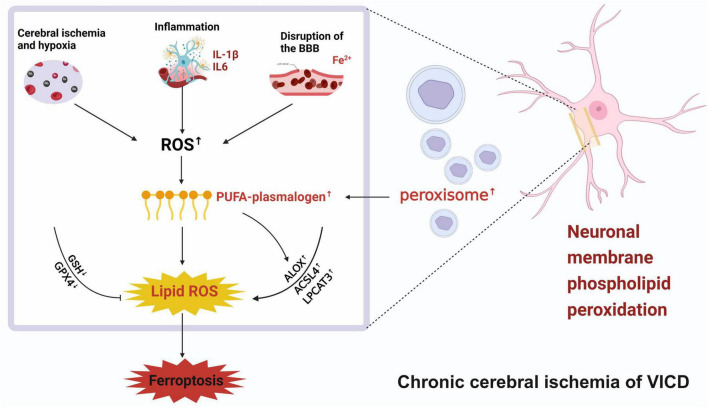
Peroxisome may promote ferroptosis of VICD. The underlying mechanism of VICD involves persistent ischemia and hypoxia, which not only directly trigger the development of a significant quantity of ROS in the brain, but also enhance ROS generation through inflammatory responses. Ischemia and hypoxia harm the small blood vessels and the BBB in the brain, leading to an excessive flow of blood into the brain tissue. The lysis of erythrocytes results in the liberation of a substantial quantity of Fe^2+^. It can undergo Fenton reaction to generate a large amount of ROS, directly promoting lipid peroxidation. It can also act as a cofactor of lipid peroxidation enzymes to promote lipid peroxidation and induce fereoptosis. Hyperlipidemia, diabetes, and atherosclerosis are some of the predisposing factors for VCID. They can disrupt intercellular metabolism, change cellular lipid composition, and elevate peroxisome and PUFA-plasmalogen, which leads to increased oxidative damage. The decrease of GSH and GPX4 within brain tissue, as well as the increase of ALOX, ACSL4, and LPCAT3, exacerbate neuronal lipid peroxidation and induce ferroptosis.

Peroxisome biogenesis factors (PEXs) are a class of proteins essential for the assembly of functional peroxisomes. These proteins collectively contribute to the biosynthesis and maintenance of peroxisomes, ensuring the normal metabolic and physiological functions of cells (Zou et al., 2020a). Among them, PEX3 and PEX10 are responsible for the localization and assembly of peroxisomal membrane proteins, playing a critical role in peroxisome formation and ensuring proper peroxisomal function. Knockout of PEX3 and PEX10 has been shown to reduce the number of peroxisomes in cells and enhance cellular resistance to ferroptosis. Meanwhile, PEX13 and PEX5 act as peroxisomal receptor import proteins and are involved in the regulation of ferroptosis sensitivity (Gao et al., 2022). These findings suggest that peroxisomes can induce ferroptosis by altering membrane phospholipid composition and reducing the threshold for lipid peroxidation in membrane phospholipids. It is further speculated that peroxisomes may promote ferroptosis in VCID through this mechanism (Figure 7).

However, a paradox exists, as plasmalogens produced by peroxisomes are also critical components of myelin, which help maintain the structural integrity and normal function of axonal membranes. In mice lacking peroxisomes, axonal degeneration, progressive demyelination, and B cell- and T cell-mediated inflammatory responses are observed, indicating the pivotal role of peroxisomes in neuroprotection. The delicate balance of peroxisomal function is further reflected in its dual role as a subcellular organelle, encompassing both lipid oxidation and antioxidant systems. It not only breaks down very long-chain fatty acids, producing hydrogen peroxide, but also utilizes its antioxidant machinery to neutralize the hydrogen peroxide generated. These studies highlight the extraordinary plasticity of peroxisomes, which can rapidly adjust their functions in response to environmental conditions and various stimuli. Under normal circumstances, peroxisomes maintain the balance between oxidative and antioxidant systems within cells to protect neurons. However, under pathological conditions, they may induce neuronal ferroptosis and consequent neurofunctional impairment by altering membrane phospholipid composition and facilitating lipid peroxidation.

Peroxisomal dysfunction and ferroptosis are intricately linked in VCID. Evidence suggests that peroxisomal impairment can predispose neurons to ferroptosis, while ferroptosis-associated oxidative damage can further exacerbate peroxisomal dysfunction (Tang and Kroemer, 2020), forming a vicious cycle that accelerates neurodegeneration. Peroxisomal dysfunction as a cause of ferroptosis. Peroxisomes play a key role in lipid metabolism, ROS detoxification, and iron homeostasis. Dysfunctional peroxisomal lipid metabolism, particularly impaired plasmalogen synthesis and β-oxidation of very-long-chain fatty acids (VLCFAs), can lead to excess lipid peroxidation, increasing the vulnerability of neurons to ferroptosis (Han et al., 2024). Additionally, defective peroxisomes fail to effectively neutralize ROS via Prx-6 and CAT, further elevating oxidative stress levels (Li Y. et al., 2022). These factors create a pro-ferroptotic intracellular environment, promoting lipid peroxidation, iron accumulation, and mitochondrial dysfunction—hallmarks of ferroptosis.

Conversely, ferroptosis itself may aggravate peroxisomal dysfunction. During ferroptosis, excessive ROS and lipid peroxidation byproducts such as MDA and 4-HNE can damage peroxisomal membranes, impairing their function (Dalleau et al., 2013). Moreover, iron overload in ferroptosis may disrupt peroxisomal protein homeostasis by affecting PEX proteins, leading to peroxisome degradation (Han et al., 2024). This creates a positive feedback loop, where ferroptosis-induced peroxisomal damage leads to further oxidative stress, thereby worsening neuroinflammation and neuronal loss in VCID.

In addition to peroxisome, mitochondria are also important organelles for lipid metabolism and redox homeostasis, and their interplay influences ferroptosis susceptibility (Guo et al., 2022). While mitochondria serve as primary hubs for energy production and oxidative phosphorylation, it function is also highly regulated by lipid metabolism. Dysfunction in peroxisomes may disrupt mitochondrial lipid metabolism, exacerbating lipid peroxidation and accelerating ferroptosis (Han et al., 2024). A key link in this interaction is plasmalogen metabolism. As antioxidant phospholipids synthesized in peroxisomes, plasmalogens shield mitochondrial membranes from oxidative damage (Zou et al., 2020a). However, peroxisomal dysfunction impairs plasmalogen synthesis, resulting in increased mitochondrial lipid peroxidation and heightened ferroptotic vulnerability.

Another crucial regulatory mechanism involves 7-DHC, a sterol metabolite recently identified as an endogenous ferroptosis suppressor (Freitas et al., 2024). With high reactivity toward peroxyl radicals, 7-DHC functions as a lipid antioxidant, reducing ferroptotic susceptibility by intercepting oxidative chain reactions. However, peroxisomal dysfunction may disrupt 7-DHC homeostasis. The latest evidence shows that ROS increases after cerebral ischemia, leading to an increase in hypoxia inducible factors, which may further damage the function of peroxisomes and disrupt the homeostasis of 7-DHC, preventing it from performing its free radical capture function on the mitochondrial inner membrane (Li Y. et al., 2024). This increases mitochondrial lipid peroxidation and makes cells susceptible to iron deposition.

Furthermore, peroxisomal β-oxidation of VLCFAs generates metabolic intermediates like acetyl-CoA and NADH, which are transferred to mitochondria for TCA cycle activity and ATP synthesis (Tang et al., 2021). Impaired peroxisomal β-oxidation disrupts mitochondrial redox balance, leading to excessive ROS accumulation and iron-driven oxidative stress, further amplifying ferroptotic signaling (Chen J. et al., 2023). Therefore, mitochondrial crosstalk of peroxisomes is another influencing factor of ferroptosis susceptibility, with plasmalogens, 7-DHC, and VLCFA β-oxidation playing central roles in regulating oxidative stress. Disruptions in this network not only compromise mitochondrial integrity but also create a self-perpetuating cycle of lipid peroxidation, reinforcing ferroptosis progression.

### 8.1 The regulatory effects of acupuncture on peroxisomes

Peroxiredoxins (Prxs) are a family of antioxidant enzymes localized within peroxisomes. Under physiological conditions, they neutralize the H_2_O_2_ produced during fatty acid breakdown in peroxisomes, thereby maintaining cellular redox balance and preventing oxidative damage. Studies have shown that the level of peroxiredoxin-6 (Prx-6) in the brain tissue of MCAO/R rats is higher than that in the sham group, but its level is downregulated after EA intervention. However, EA preconditioning can increase the expression of Prx-6 in the brain tissue of MCAO/R rats, thereby exerting neuroprotective effects (Gao et al., 2014). The above results suggest that EA has the potential to regulate peroxisomes.

Existing evidence also indicates that acupuncture can modulate the expression of peroxisome proliferator-activated receptor-γ (PPARγ) proteins and genes. Studies have shown that EA intervention at Chize (LU5), Hegu (LI4), Sanyinjiao (SP6), and ST36 of the MCAO rats can upgrade the total expression of PPARγprotein, alleviating neuroinflammation in the brain tissue post-stroke, thereby improving neurological function scores in rats (Yao et al., 2023). Furthermore, acupuncture at GV20 and BL23 may have beneficial effects on cognitive impairment in AD rats by activating PPAR-γ and inhibiting the expression of p-p38MAPK (Zhang et al., 2017). EA can also improve cognitive function and motor abilities in cerebral palsy rat models by activating the PPAR pathway (Wu Z.-F. et al., 2024).

Peroxisomes exhibit a dual role in ferroptosis and neuroprotection, primarily through their involvement in plasmalogen synthesis and antioxidant defense mechanisms. On one hand, peroxisomes produce plasmalogens, a class of ether phospholipids that are highly susceptible to lipid peroxidation, thus making cells more vulnerable to ferroptosis. On the other hand, peroxisomes are critical for myelin maintenance, VLCFA metabolism, and ROS detoxification, all of which contribute to neuronal resilience (Cui W. et al., 2021). Acupuncture appears to modulate peroxisomal activity by fine-tuning the balance between these competing roles, thereby preventing excessive ferroptotic damage while preserving myelin integrity.

There are studies suggesting that acupuncture enhances peroxisomal antioxidant defense systems by upregulating Prx-6, a crucial peroxisomal enzyme involved in lipid peroxidation repair and ROS scavenging (Gao et al., 2014). Increased Prx-6 activity has been associated with reduced oxidative damage in neuronal membranes and enhanced protection against ferroptosis-induced demyelination. Furthermore, acupuncture has been shown to modulate PPARγ signaling, a key regulator of peroxisomal function that governs lipid metabolism, anti-inflammatory responses, and neuroprotection (Yao et al., 2023). Activation of PPARγ by acupuncture may support myelin regeneration and reduce peroxisome-mediated lipid peroxidation, thereby preventing ferroptosis while sustaining neuronal function.

Moreover, acupuncture appears to influence peroxisome biogenesis through the regulation of PEX proteins, which are essential for maintaining peroxisomal integrity and function (Gao et al., 2014). Acupuncture may promote the expression of PEXs family, thereby supporting peroxisomal homeostasis and ensuring a controlled production of plasmalogens that does not exacerbate ferroptosis. By regulating peroxisomal functions in a targeted and adaptive manner, acupuncture provides a neuroprotective strategy that mitigates ferroptotic vulnerability without impairing essential lipid metabolism and myelin maintenance. These findings highlight acupuncture’s potential as a dual-action intervention, offering both ferroptosis inhibition and structural support for neuronal health in VCID.

## 9 Summary and prospect

Description of ferroptosis as a unique cell death modality, closely associated with VCID (Fu et al., 2023; Yan and Zhang, 2019), involves complex etiological factors (Hu et al., 2024), pathological manifestations (Liu et al., 2015; Sun et al., 2017), and mechanisms (David et al., 2023; Figure 2). As research on ferroptosis advances, we are beginning to understand how this network plays a critical role in the pathophysiology of VCID, encompassing the dysregulation of glycolipid metabolism, aberrations in glutathione metabolism, disturbance of iron homeostasis, and an imbalance in redox reactions, as well as the regulatory effect of acupuncture on them (Lang et al., 2024).

First, we share some concerns and unresolved questions related to ferroptosis in VCID. One of the core mechanisms of ferroptosis lies in the complex interplay between intracellular iron metabolism and lipid peroxidation (Tian et al., 2024). Peroxisomes, as key regulators of lipid metabolism within cells, play a crucial role not only in maintaining normal cellular functions but also in modulating the sensitivity of cells to ferroptosis under pathological conditions by regulating the production of plasmalogens (Cui W. et al., 2021; Tang and Kroemer, 2020). Our review suggests that acupuncture may indirectly influence the lipid peroxidation process by modulating peroxisomal functions, thereby inhibiting ferroptosis and improving neurological function in VCID patients. This perspective not only provides a new avenue for the clinical application of acupuncture but also opens up new research directions for exploring the role of peroxisomes in neurodegenerative diseases (Li J. et al., 2022). However, the role of peroxisomes in ferroptosis is dualistic. On one hand, peroxisomes contribute to the production of plasmalogens, which increase the susceptibility of cell membranes to oxidative damage, potentially exacerbating ferroptosis (Tang and Kroemer, 2020). On the other hand, peroxisomes are integral to the lipid oxidative degradation and antioxidant systems, capable of maintaining cellular oxidative balance by breaking down very long-chain fatty acids and scavenging hydrogen peroxide (Tang et al., 2023). This duality complicates the role of peroxisomes in ferroptosis, and whether acupuncture can precisely modulate this balance to achieve therapeutic effects remains to be thoroughly investigated. We propose a future research direction based on this. Future research should explore the precise role of peroxisomes in ferroptosis across different neurodegenerative conditions, including VCID, AD, and PD, to determine whether their contribution to oxidative stress outweighs their protective antioxidant functions.

Second, we note some challenges. Current studies suggest that acupuncture may exert its neuroprotection effects by modulating signaling pathways such as the Prx-6 and PPARγ, but the specific mechanisms remain unclear and the range of conditions it can regulate appears to be limited (Wu Z.-F. et al., 2024). In particular, there is a lack of deep research on peroxisomes and ferroptosis in the treatment of VICD with acupuncture and moxibustion. Given the complexity of peroxisomal and ferroptosis pathways, relying on the regulation of a single signaling pathway may be insufficient to achieve the desired therapeutic outcomes. Therefore, future acupuncture research should focus on the multi-target and multi-pathway regulatory effects of peroxisomes and ferroptosis in the treatment of VCID, while also exploring the potential applications of acupuncture in more complex pathological conditions.

Furthermore, the variability in acupuncture techniques poses significant challenges for generalizing results across studies and patient populations. We acknowledge that the variability in acupuncture techniques is a double-edged sword, presenting both advantages and challenges in research generalization. On one hand, acupuncture is inherently adaptable, requiring personalized prescriptions tailored to the specific disease and individual patient conditions. This adaptability allows acupuncture to serve as a versatile adjunct therapy, complementing standard treatments and facilitating patient recovery across a wide range of diseases. However, this variability also introduces challenges, as the same disease and patient may receive slightly different acupuncture treatments depending on the practitioner, potentially leading to inconsistent therapeutic outcomes. To enhance reproducibility and generalizability, it is crucial to strengthen training for acupuncture practitioners and establish standardized acupuncture prescriptions for specific diseases. These guidelines should include acupoint selection, needle insertion techniques, stimulation frequency, duration, and intensity, ensuring that clinical acupuncture practices are both effective and scientifically rigorous. Additionally, incorporating standardized outcome measures, such as biomarkers of ferroptosis, neuroimaging, and cognitive assessments, will further facilitate cross-study comparisons and meta-analyses (Reichert et al., 2020). In animal studies, where acupuncture is frequently used to investigate mechanistic pathways, adopting standardized protocols is essential to minimize procedural variability. By refining both clinical and experimental acupuncture methodologies, we can enhance the reliability of research findings and improve the consistency of acupuncture applications across diverse patient populations.

Besides, to objectively evaluate acupuncture’s role in ferroptosis regulation, incorporating reliable biomarkers into clinical trials is crucial. Several potential serum-based biomarkers can be used to track lipid peroxidation and iron metabolism changes, including plasmalogens, lipid peroxidation markers (such as MDA and 4-HNE), and ferroptosis-related proteins like ACSL4 and GPX4. These markers provide insight into lipid oxidation status, antioxidant enzyme activity, and iron homeostasis, making them valuable tools for assessing acupuncture’s therapeutic effects. However, when studying central nervous system (CNS) disorders such as VCID, serum biomarkers alone may not fully reflect ferroptotic changes occurring in the brain. Due to the BBB, peripheral blood samples may not accurately represent lipid metabolic alterations within the CNS. To address this limitation, cerebrospinal fluid (CSF) analysis could offer a more direct assessment of brain-specific ferroptosis markers (Ryan et al., 2024). However, CSF collection is invasive, costly, and poses ethical challenges, often leading to patient reluctance and practical barriers in clinical research. Given these limitations, animal studies provide an optimal model for tracking ferroptosis-related biomarkers in both blood and brain tissue. By integrating CSF analysis in preclinical research, we can establish a clearer link between peripheral and central ferroptosis-related markers, refining our understanding of acupuncture’s neuroprotective mechanisms. Future studies should explore correlations between serum and CNS ferroptosis biomarkers, enabling less invasive yet reliable clinical assessments of acupuncture’s efficacy in regulating ferroptosis and promoting neurological health.

Last, we can predict some potential future discoveries that lie ahead in the field of ferroptosis. In terms of clinical translation, although the application of acupuncture in VCID holds great promise, its actual efficacy still requires validation through large-scale clinical trials. Issues such as individual variability in response to acupuncture, the duration of its effects, and potential side effects also warrant further investigation. Moreover, the integration of modern technologies—such as imaging, molecular biology, and multi-omics analysis—will be crucial in elucidating the effects of acupuncture on ferroptosis and its related pathways, representing a key direction for future research. Given that acupuncture’s ability to regulate ferroptosis-related pathways may involve gene transcription activation preceding molecular changes, particularly through epigenetic modifications, further investigation into these mechanisms is warranted (Luobin et al., 2023). Evidence suggests that acupuncture can regulate DNA methylation in cortical regions, including the prefrontal cortex, anterior cingulate cortex, and primary somatosensory cortex, which are associated with mitochondrial dysfunction, oxidative phosphorylation, and inflammatory pathways (Jang et al., 2024). Additionally, acupuncture has been shown to modulate DNA methylation in hippocampal neurons, influencing genes related to neurogenesis and synaptic plasticity (Jang et al., 2024). These findings highlight the potential for acupuncture to exert long-term regulatory effects at the epigenetic level, providing a promising avenue for future research on acupuncture-based interventions for ferroptosis inhibition in VCID.

Currently, there are limited clinical trials specifically targeting ferroptosis in VCID. However, ferroptosis inhibition is actively being investigated in related neurodegenerative diseases, which may provide valuable insights applicable to VCID. A recent review identified 16 clinical trials associated with ferroptosis, underscoring the growing research focus on therapeutic strategies for iron-dependent lipid peroxidation (Alves et al., 2025). These ongoing trials primarily explore ferroptosis inhibition in Alzheimer’s disease (Ayton et al., 2015, 2018), Parkinson’s disease (Bi et al., 2020; Peng et al., 2010), and ischemic stroke (Enomoto et al., 2019; Zhou et al., 2022), which share common oxidative stress and neuroinflammatory pathways with VCID.

In summary, this review delves deeply into the potential mechanisms of ferroptosis in VCID, with a particular focus on the significant roles of peroxisomes and plasmalogens in ferroptosis. We highlight the therapeutic potential of acupuncture, a non-pharmacological intervention, in modulating ferroptosis-related pathways for the treatment of VCID. Although the specific mechanisms underlying acupuncture’s effects remain incompletely understood, existing research provides a meaningful reference for further exploration of its potential in regulating ferroptosis.

## References

[B1] Abdalkader M. Lampinen R. Kanninen K. M. Malm T. M. Liddell J. R. (2018). Targeting Nrf2 to suppress ferroptosis and mitochondrial dysfunction in neurodegeneration. *Front. Neurosci.* 12:466. 10.3389/fnins.2018.00466 30042655 PMC6048292

[B2] Abe M. Sou M. Matsuoka Y. Morimoto K. Yamada K.-I. (2024). Ethoxyquin, a lipid peroxidation inhibitor, has protective effects against white matter lesions in a mouse model of chronic cerebral hypoperfusion. *Biol. Pharm. Bull.* 47 104–111. 10.1248/bpb.b23-00538 38171771

[B3] Allegra S. Comità S. Roetto A. De Francia S. (2024). Sex and gender differences in iron chelation. *Biomedicines* 12:12. 10.3390/biomedicines12122885 39767791 PMC11673655

[B4] Altamura S. Kopf S. Schmidt J. Müdder K. da Silva A. R. Nawroth P. (2017). Uncoupled iron homeostasis in type 2 diabetes mellitus. *J. Mol. Med.* 95 1387–1398. 10.1007/s00109-017-1596-3 28971221

[B5] Alves F. Lane D. Nguyen T. P. M. Bush A. I. Ayton S. (2025). In defence of ferroptosis. *Signal Transduction Targeted Ther.* 10 1–29. 10.1038/s41392-024-02088-5 39746918 PMC11696223

[B6] Appleton J. P. Scutt P. Sprigg N. Bath P. M. (2017). Hypercholesterolaemia and vascular dementia. *Clin. Sci.* 131 1561–1578. 10.1042/CS20160382 28667059

[B7] Ayton S. Diouf I. Bush A. I. Alzheimer’s, disease Neuroimaging, and Initiative. (2018). Evidence that iron accelerates Alzheimer’s pathology: A CSF biomarker study. *J. Neurol. Neurosurg. Psychiatry* 89 456–460. 10.1136/jnnp-2017-316551 28939683

[B8] Ayton S. Faux N. G. Bush A. I. Alzheimer’s Disease, and Neuroimaging Initiative. (2015). Ferritin levels in the cerebrospinal fluid predict Alzheimer’s disease outcomes and are regulated by APOE. *Nat. Commun.* 6:6760. 10.1038/ncomms7760 25988319 PMC4479012

[B9] Bai T. Li M. Liu Y. Qiao Z. Wang Z. (2020). Inhibition of ferroptosis alleviates atherosclerosis through attenuating lipid peroxidation and endothelial dysfunction in mouse aortic endothelial cell. *Free Radical Biol. Med.* 160 92–102. 10.1016/j.freeradbiomed.2020.07.026 32768568

[B10] Belavgeni A. Meyer C. Stumpf J. Hugo C. Linkermann A. (2020). Ferroptosis and necroptosis in the kidney. *Cell Chem. Biol.* 27 448–462. 10.1016/j.chembiol.2020.03.016 32302582

[B11] Bi M. Du X. Jiao Q. Liu Z. Jiang H. (2020). α-Synuclein regulates iron homeostasis via preventing parkin-mediated DMT1 Ubiquitylation in Parkinson’s disease models. *ACS Chem. Neurosci.* 11 1682–1691. 10.1021/acschemneuro.0c00196 32379419

[B12] Biessels G. J. Despa F. (2018). Cognitive decline and dementia in diabetes mellitus: Mechanisms and clinical implications. *Nat. Rev. Endocrinol.* 14 591–604. 10.1038/s41574-018-0048-7 30022099 PMC6397437

[B13] Brass S. D. Chen N. Mulkern R. V. Bakshi R. (2006). Magnetic resonance imaging of iron deposition in neurological disorders. *Topics Magn. Reson. Imaging* 17 31–40. 10.1097/01.rmr.0000245459.82782.e4 17179895

[B14] Breteler M. M. Claus J. J. Grobbee D. E. Hofman A. (1994). Cardiovascular disease and distribution of cognitive function in elderly people: The Rotterdam Study. *BMJ* 308 1604–1608. 10.1136/bmj.308.6944.1604 8025427 PMC2540432

[B15] Cao Y. Murphy K. J. McIntyre T. M. Zimmerman G. A. Prescott S. M. (2000). Expression of fatty acid-CoA ligase 4 during development and in brain. *FEBS Lett.* 467 263–267. 10.1016/s0014-5793(00)01159-5 10675551

[B16] Cedillo L. Ahsan F. M. Li S. Stuhr N. L. Zhou Y. Zhang Y. (2023). Ether lipid biosynthesis promotes lifespan extension and enables diverse pro-longevity paradigms in Caenorhabditis elegans. *eLife* 12:e82210. 10.7554/eLife.82210 37606250 PMC10444025

[B17] Chan K. Y. Wang W. Wu J. J. Liu L. Theodoratou E. Car J. (2013). Epidemiology of Alzheimer’s disease and other forms of dementia in China, 1990-2010: A systematic review and analysis. *Lancet* 381 2016–2023. 10.1016/S0140-6736(13)60221-4 23746902

[B18] Chang Wong E. Chang Chui H. (2022). Vascular cognitive impairment and dementia. *Continuum* 28 750–780. 10.1212/CON.0000000000001124 35678401 PMC9833847

[B19] Chang Q.-Y. Lin Y.-W. Hsieh C.-L. (2018). Acupuncture and neuroregeneration in ischemic stroke. *Neural Regeneration Res.* 13:573. 10.4103/1673-5374.230272 29722298 PMC5950656

[B20] Chang S. Liu J. Wen J. Li H. Dong G. Liu C. (2023). The impact of multiple risk factors for cognitive impairment on the pathological morphology and iron content of brain tissue in hypertensive rats. *Acta Lab. Anim. Sci. Sin.* 31 706–714.

[B21] Chaudhary N. Pandey A. S. Griauzde J. Gemmete J. J. Chenevert T. L. Keep R. F. (2019). Brain tissue iron quantification by MRI in intracerebral hemorrhage: Current translational evidence and pitfalls. *J. Cereb. Blood Flow Metab.* 39 562–564. 10.1177/0271678X18818911 30540218 PMC6421244

[B22] Chen A. Lin Z. Lan L. Xie G. Huang J. Lin J. (2012). Electroacupuncture at the Quchi and Zusanli acupoints exerts neuroprotective role in cerebral ischemia-reperfusion injured rats via activation of the PI3K/Akt pathway. *Int. J. Mol. Med.* 30 791–796. 10.3892/ijmm.2012.1074 22842715

[B23] Chen C.-H. Hsieh C.-L. (2020). Effect of acupuncture on oxidative stress induced by cerebral ischemia-reperfusion injury. *Antioxidants* 9:3. 10.3390/antiox9030248 32204376 PMC7139408

[B24] Chen H. Wu C. Lv Q. Li M. Ren L. (2023). Targeting mitochondrial homeostasis: The role of acupuncture in depression treatment. *Neuropsychiatric Dis. Treatment* 19 1741–1753. 10.2147/NDT.S421540 37546517 PMC10404048

[B25] Chen J. Wu K. Lei Y. Huang M. Cheng L. Guan H. (2023). Inhibition of fatty acid β-Oxidation by fatty acid binding protein 4 induces ferroptosis in HK2 cells under high glucose conditions. *Endocrinol. Metab.* 38 226–244. 10.3803/EnM.2022.1604 37150518 PMC10164503

[B26] Chen K. Jiang X. Wu M. Cao X. Bao W. Zhu L.-Q. (2021a). Ferroptosis, a potential therapeutic target in Alzheimer’s disease. *Front. Cell Dev. Biol.* 9:704298. 10.3389/fcell.2021.704298 34422824 PMC8374166

[B27] Chen Q. Song W. Tang Y. Tang Y. Kang Y. Zhu L. (2022). Electroacupuncture reduces cerebral hemorrhage injury in rats by improving cerebral iron metabolism. *Mediators Inflammation* 2022:6943438. 10.1155/2022/6943438 36016663 PMC9398869

[B28] Chen T. Zhang B. Zhang X. Tang L. Wang C. (2025). Electroacupuncture improves postoperative cognitive dysfunction by inhibiting ferroptosis via the TFR1-DMT1-FPN pathway. *Acupuncture Med.* 10.1177/09645284241302816 Online ahead of print. 39754452

[B29] Chen X. Kang R. Kroemer G. Tang D. (2021b). Organelle-specific regulation of ferroptosis. *Cell Death Diff.* 28 2843–2856. 10.1038/s41418-021-00859-z 34465893 PMC8481335

[B30] Chen X. Li J. Kang R. Klionsky D. J. Tang D. (2021c). Ferroptosis: Machinery and regulation. *Autophagy* 17 2054–2081. 10.1080/15548627.2020.1810918 32804006 PMC8496712

[B31] Chen Z. Sun X. Li X. Liu N. (2023). Oleoylethanolamide alleviates hyperlipidaemia-mediated vascular calcification via attenuating mitochondrial DNA stress triggered autophagy-dependent ferroptosis by activating PPARα. *Biochem. Pharmacol.* 208:115379. 10.1016/j.bcp.2022.115379 36525991

[B32] Chi S. I. Wang C. K. Chen J. J. Chau L. Y. Lin T. N. (2000). Differential regulation of H- and L-ferritin messenger RNA subunits, ferritin protein and iron following focal cerebral ischemia-reperfusion. *Neuroscience* 100 475–484. 10.1016/s0306-4522(00)00317-1 11098110

[B33] Cui W. Liu D. Gu W. Chu B. (2021). Peroxisome-driven ether-linked phospholipids biosynthesis is essential for ferroptosis. *Cell Death Diff.* 28 2536–2551. 10.1038/s41418-021-00769-0 33731874 PMC8329287

[B34] Cui Y. Zhang Y. Zhao X. Shao L. Liu G. Sun C. (2021). ACSL4 exacerbates ischemic stroke by promoting ferroptosis-induced brain injury and neuroinflammation. *Brain Behav. Immun.* 93 312–321. 10.1016/j.bbi.2021.01.003 33444733

[B35] Dai X. Zhang H. Yu W. Zhao Y. (2024). Exploring the regulatory effect of acupuncture on iron death in brain tissue after cerebral hemorrhage based on the p62/Keap1/NRF2 signaling pathway. *Inf. Tradit. Chin. Med.* 41 44–50. 10.19656/j.cnki.1002-2406.20240208

[B36] Dalleau S. Baradat M. Guéraud F. Huc L. (2013). Cell death and diseases related to oxidative stress:4-hydroxynonenal (HNE) in the balance. *Cell Death Diff.* 20 1615–1630. 10.1038/cdd.2013.138 24096871 PMC3824598

[B37] Dang Y. He Q. Yang S. Sun H. Liu Y. Li W. (2022). FTH1- and SAT1-induced astrocytic ferroptosis is involved in Alzheimer’s Disease: Evidence from single-cell transcriptomic analysis. *Pharmaceuticals* 15:10. 10.3390/ph15101177 36297287 PMC9610574

[B38] Das S. K. Patel V. B. Basu R. Wang W. DesAulniers J. Kassiri Z. (2017). Females are protected from iron-overload cardiomyopathy independent of iron metabolism: Key role of oxidative stress. *J. Am. Heart Assoc.* 6:e003456. 10.1161/JAHA.116.003456 28115312 PMC5523622

[B39] David S. Ryan F. Jhelum P. Kroner A. (2023). Ferroptosis in neurological disease. *Neuroscientist* 29 591–615. 10.1177/10738584221100183 35678019

[B40] DeGregorio-Rocasolano N. Martí-Sistac O. Gasull T. (2019). Deciphering the iron side of stroke: Neurodegeneration at the crossroads between iron dyshomeostasis, excitotoxicity, and ferroptosis. *Front. Neurosci.* 13:85. 10.3389/fnins.2019.00085 30837827 PMC6389709

[B41] Demougeot C. Van Hoecke M. Bertrand N. Prigent-Tessier A. Mossiat C. Beley A. (2004). Cytoprotective efficacy and mechanisms of the liposoluble iron chelator 2,2′-dipyridyl in the rat photothrombotic ischemic stroke model. *J. Pharmacol. Exp. Therapeutics* 311 1080–1087. 10.1124/jpet.104.072744 15280435

[B42] Dhuriya Y. K. Sharma D. (2018). Necroptosis: A regulated inflammatory mode of cell death. *J. Neuroinflammation* 15 199. 10.1186/s12974-018-1235-0 29980212 PMC6035417

[B43] Dietrich R. B. Bradley W. G. (1988). Iron accumulation in the basal ganglia following severe ischemic-anoxic insults in children. *Radiology* 168 203–206. 10.1148/radiology.168.1.3380958 3380958

[B44] Ding H. Yan C.-Z. Shi H. Zhao Y.-S. Chang S.-Y. Yu P. (2011). Hepcidin is involved in iron regulation in the ischemic brain. *PLoS One* 6:e25324. 10.1371/journal.pone.0025324 21957487 PMC3177902

[B45] Ding L. Teng R. Zhu Y. Liu F. Wu L. Qin L. (2023). Electroacupuncture treatment ameliorates metabolic disorders in obese ZDF rats by regulating liver energy metabolism and gut microbiota. *Front. Endocrinol.* 14:1207574. 10.3389/fendo.2023.1207574 37441502 PMC10335763

[B46] Ding X.-S. Gao L. Han Z. Eleuteri S. Shi W. Shen Y. (2023). Ferroptosis in Parkinson’s disease: Molecular mechanisms and therapeutic potential. *Ageing Res. Rev.* 91:102077. 10.1016/j.arr.2023.102077 37742785

[B47] Dixon S. J. Lemberg K. M. Lamprecht M. R. Skouta R. Zaitsev E. M. Gleason C. E. (2012). Ferroptosis: An iron-dependent form of nonapoptotic cell death. *Cell* 149 1060–1072. 10.1016/j.cell.2012.03.042 22632970 PMC3367386

[B48] Doll S. Proneth B. Tyurina Y. Y. Panzilius E. Kobayashi S. Ingold I. (2017). ACSL4 dictates ferroptosis sensitivity by shaping cellular lipid composition. *Nat. Chem. Biol.* 13 91–98. 10.1038/nchembio.2239 27842070 PMC5610546

[B49] Drgová A. Likavcanová K. Dobrota D. (2004). Changes of phospholipid composition and superoxide dismutase activity during global brain ischemia and reperfusion in rats. *General Physiol. Biophys.* 23 337–346. 15638121

[B50] Du S. Wang X. Zhu W. Ji C. Li T. Xiao L. (2017). Effects of acupuncture on oxidative stress and neuronal damage in prefrontal cortex of vascular dementia rats. *Chin. J. Inf. Tradit. Chin. Med.* 24 53–55.

[B51] Du S.-Q. Wang X.-R. Xiao L.-Y. Tu J.-F. Zhu W. He T. (2017). Molecular mechanisms of vascular dementia: What can be learned from animal models of chronic cerebral hypoperfusion? *Mol. Neurobiol.* 54 3670–3682. 10.1007/s12035-016-9915-1 27206432

[B52] Du W. Wang Z. Dong Y. Hu H. Zhou H. He X. (2023). Electroacupuncture promotes skin wound repair by improving lipid metabolism and inhibiting ferroptosis. *J. Cell. Mol. Med.* 27 2308–2320. 10.1111/jcmm.17811 37307402 PMC10424292

[B53] Edgerton-Fulton M. Ergul A. (2022). Vascular contributions to cognitive impairment/dementia in diabetes: Role of endothelial cells and pericytes. *Am. J. Physiol. Cell Physiol.* 323 C1177–C1189. 10.1152/ajpcell.00072.2022 36036445 PMC9576164

[B54] Elmore S. (2007). Apoptosis: A review of programmed cell death. *Toxicol. Pathol.* 35 495–516. 10.1080/01926230701320337 17562483 PMC2117903

[B55] Enomoto M. Endo A. Yatsushige H. Fushimi K. Otomo Y. (2019). Clinical effects of early edaravone use in acute ischemic stroke patients treated by endovascular reperfusion therapy. *Stroke* 50 652–658. 10.1161/STROKEAHA.118.023815 30741623

[B56] Faber C. L. Deem J. D. Campos C. A. Taborsky G. J. Morton G. J. (2020). CNS control of the endocrine pancreas. *Diabetologia* 63 2086–2094. 10.1007/s00125-020-05204-6 32894319 PMC7983553

[B57] Fan B.-Y. Pang Y.-L. Li W.-X. Zhao C.-X. Zhang Y. Wang X. (2020). Liproxstatin-1 is an effective inhibitor of oligodendrocyte ferroptosis induced by inhibition of glutathione peroxidase 4. *Neural Regeneration Res.* 16 561–566. 10.4103/1673-5374.293157 32985488 PMC7996026

[B58] Fang K.-M. Cheng F.-C. Huang Y.-L. Chung S.-Y. Jian Z.-Y. Lin M.-C. (2013). Trace element, antioxidant activity, and lipid peroxidation levels in brain cortex of gerbils after cerebral ischemic injury. *Biol. Trace Element Res.* 152 66–74. 10.1007/s12011-012-9596-1 23334863

[B59] Fang X. Ardehali H. Min J. Wang F. (2023). The molecular and metabolic landscape of iron and ferroptosis in cardiovascular disease. *Nat. Rev. Cardiol.* 20 7–23. 10.1038/s41569-022-00735-4 35788564 PMC9252571

[B60] Fei Y. Ding Y. (2024). The role of ferroptosis in neurodegenerative diseases. *Front. Cell. Neurosci.* 18:1475934. 10.3389/fncel.2024.1475934 39473490 PMC11518764

[B61] Freitas F. P. Alborzinia H. Dos Santos A. F. Nepachalovich P. Pedrera L. Zilka O. (2024). 7-Dehydrocholesterol is an endogenous suppressor of ferroptosis. *Nature* 626 401–410. 10.1038/s41586-023-06878-9 38297129

[B62] Fu P. Chen Y. Wu M. Bao B. Yin X. Chen Z. (2023). Effect of ferroptosis on chronic cerebral hypoperfusion in vascular dementia. *Exp. Neurol.* 370:114538. 10.1016/j.expneurol.2023.114538 37709116

[B63] Galluzzi L. Vitale I. Aaronson S. A. Abrams J. M. Adam D. Agostinis P. (2018). Molecular mechanisms of cell death: Recommendations of the nomenclature committee on cell death 2018. *Cell Death Diff.* 25 486–541. 10.1038/s41418-017-0012-4 29362479 PMC5864239

[B64] Gao Y. (2023). *Buyang Huanwu Decoction Alleviated Inflammatory Injury After Cerebral Ischemia-Reperfusion in Rats via Activating APN Pathway to inhibit 12/15-LOX.* Master’s thesis. Guangzhou: Guangzhou University of Traditional Chinese Medicine, 10.27044/d.cnki.ggzzu.2023.000542

[B65] Gao Y. Yang J. (2023). The effect of awakening the brain and opening the orifice acupuncture method on neuronal ferroptosis in hemorrhagic stroke rats. *J. Guangxi. Univ. Chin. Med.* 26 40–45.

[B66] Gao Y. Li J. Su M. Li Y. (2017). [Acupuncture with smoothing liver and regulating qi for post-stroke slow transit constipation and its gastrointestinal hormone level]. *Chin. Acupuncture Moxibustion* 37 125–129. 10.13703/j.0255-2930.2017.02.005 29231472

[B67] Gao Y. Liang C. Zhang Q. Zhuang H. Sui C. Zhang N. (2025). Brain iron deposition and cognitive decline in patients with cerebral small vessel disease: A quantitative susceptibility mapping study. *Alzheimer’s Res. Therapy* 17:17. 10.1186/s13195-024-01638-x 39789638 PMC11715900

[B68] Gao Y. Skowyra M. L. Feng P. Rapoport T. A. (2022). Protein import into peroxisomes occurs through a nuclear pore-like phase. *Science* 378:eadf3971. 10.1126/science.adf3971 36520918 PMC9795577

[B69] Gao Y. Sun S. Liu X. Li L. Zhang Z. Chen S. (2014). Electroacupuncture pretreatment attenuated focal cerebral ischemia/ reperfusioniniury by up egulation of peroxiredoxin 6 in rates. *Chin. J. Neuroanat.* 30 507–513. 10.11670/1000-7547.201405001

[B70] Gorelick P. B. Scuteri A. Black S. E. Decarli C. Greenberg S. M. Iadecola C. (2011). Vascular contributions to cognitive impairment and dementia: A statement for healthcare professionals from the american heart association/american stroke association. *Stroke* 42 2672–2713. 10.1161/STR.0b013e3182299496 21778438 PMC3778669

[B71] Gu L. Chen H. Geng R. Sun M. Shi Q. Chen Y. (2024). Single-cell and spatial transcriptomics reveals ferroptosis as the most enriched programmed cell death process in hemorrhage stroke-induced oligodendrocyte-mediated white matter injury. *Int. J. Biol. Sci.* 20 3842–3862. 10.7150/ijbs.96262 39113700 PMC11302879

[B72] Gu Z. (2023). *Correlation Analysis of Ferroptosis Markers with Clinical Traitsand White Matter Changes in Patients with Vascular Cognitive Impairment.* Master’s thesis. Shanghai: PLA Naval Medical University, 10.26998/d.cnki.gjuyu.2022.000106

[B73] Gubern C. Camós S. Ballesteros I. Rodríguez R. Romera V. G. Cañadas R. (2013). miRNA expression is modulated over time after focal ischaemia: Up-regulation of miR-347 promotes neuronal apoptosis. *FEBS J.* 280 6233–6246. 10.1111/febs.12546 24112606

[B74] Guo J. Zhou Y. Liu D. Wang M. Wu Y. Tang D. (2022). Mitochondria as multifaceted regulators of ferroptosis. *Life Metab.* 1 134–148. 10.1093/lifemeta/loac035 39872359 PMC11749789

[B75] Gustaw-Rothenberg K. Kowalczuk K. Stryjecka-Zimmer M. (2010). Lipids’ peroxidation markers in Alzheimer’s disease and vascular dementia. *Geriatr. Gerontol. Int.* 10 161–166. 10.1111/j.1447-0594.2009.00571.x 20446930

[B76] Han J. Guo X. Meng X.-J. Zhang J. Yamaguchi R. Motoo Y. (2020). Acupuncture improved lipid metabolism by regulating intestinal absorption in mice. *World J. Gastroenterol.* 26 5118–5129. 10.3748/wjg.v26.i34.5118 32982113 PMC7495030

[B77] Han J. Zheng D. Liu P.-S. Wang S. Xie X. (2024). Peroxisomal homeostasis in metabolic diseases and its implication in ferroptosis. *Cell Commun. Signaling* 22:475. 10.1186/s12964-024-01862-w 39367496 PMC11451054

[B78] Hanson L. R. Roeytenberg A. Martinez P. M. Coppes V. G. Sweet D. C. Rao R. J. (2009). Intranasal deferoxamine provides increased brain exposure and significant protection in rat ischemic stroke. *J. Pharmacol. Exp. Therapeutics* 330 679–686. 10.1124/jpet.108.149807 19509317 PMC2729791

[B79] Hao S. Liang B. Huang Q. Dong S. Wu Z. He W. (2018). Metabolic networks in ferroptosis. *Oncol. Lett.* 15 5405–5411. 10.3892/ol.2018.8066 29556292 PMC5844144

[B80] He L. Liu Y.-Y. Wang K. Li C. Zhang W. Li Z.-Z. (2021). Tanshinone IIA protects human coronary artery endothelial cells from ferroptosis by activating the NRF2 pathway. *Biochem. Biophys. Res. Commun.* 575 1–7. 10.1016/j.bbrc.2021.08.067 34454174

[B81] He Z. Zhong W. Gao X. Song J. (2023). The redox interaction between peroxisomes and mitochondria and its research progress in diseases. *Chin. J. Mod. Doctor* 61 99–103. 10.3969/j.issn.1673-9701.2023.07.024

[B82] Henning Y. Blind U. S. Larafa S. Matschke J. Fandrey J. (2022). Hypoxia aggravates ferroptosis in RPE cells by promoting the Fenton reaction. *Cell Death Dis.* 13:662. 10.1038/s41419-022-05121-z 35906211 PMC9338085

[B83] Hou L. Sun F. Sun W. Zhang L. Wang Q. (2019). Lesion of the locus coeruleus damages learning and memory performance in paraquat and maneb-induced mouse Parkinson’s disease model. *Neuroscience* 419 129–140. 10.1016/j.neuroscience.2019.09.006 31634513

[B84] Hou W. Xie Y. Song X. Sun X. Lotze M. T. Zeh H. J. (2016). Autophagy promotes ferroptosis by degradation of ferritin. *Autophagy* 12 1425–1428. 10.1080/15548627.2016.1187366 27245739 PMC4968231

[B85] Hu X. Bao Y. Li M. Zhang W. Chen C. (2024). The role of ferroptosis and its mechanism in ischemic stroke. *Exp. Neurol.* 372:114630. 10.1016/j.expneurol.2023.114630 38056585

[B86] Huang Y.-T. Hong F.-F. Yang S.-L. (2021). Atherosclerosis: The culprit and Co-victim of vascular dementia. *Front. Neurosci.* 15:673440. 10.3389/fnins.2021.673440 34421513 PMC8377286

[B87] Huo T. Jia Y. Yin C. Luo X. Zhao J. Wang Z. (2019). Iron dysregulation in vascular dementia: Focused on the AMPK/autophagy pathway. *Brain Res. Bull.* 153 305–313. 10.1016/j.brainresbull.2019.09.006 31542426

[B88] Huo Z.-J. Li Q. Tian G.-H. Zhou C.-M. Wei X.-H. Pan C.-S. (2014). The ameliorating effects of long-term electroacupuncture on cardiovascular remodeling in spontaneously hypertensive rats. *BMC Complementary Alternative Med.* 14:118. 10.1186/1472-6882-14-118 24685050 PMC3994235

[B89] Iadecola C. Duering M. Hachinski V. Joutel A. Pendlebury S. T. Schneider J. A. (2019). Vascular cognitive impairment and dementia: JACC scientific expert panel. *J. Am. Coll. Cardiol.* 73 3326–3344. 10.1016/j.jacc.2019.04.034 31248555 PMC6719789

[B90] Ida M. Mizunuma K. Hata Y. Tada S. (1994). Subcortical low intensity in early cortical ischemia. *Am. J. Neuroradiol.* 15 1387–1393. 7526671 PMC8332443

[B91] Ide S. Ide K. Abe K. Kobayashi Y. Kitai H. McKey J. (2022). Sex differences in resilience to ferroptosis underlie sexual dimorphism in kidney injury and repair. *Cell Rep.* 41:111610. 10.1016/j.celrep.2022.111610 36351395 PMC9795409

[B92] Inoue Y. Shue F. Bu G. Kanekiyo T. (2023). Pathophysiology and probable etiology of cerebral small vessel disease in vascular dementia and Alzheimer’s disease. *Mol. Neurodegeneration* 18:46. 10.1186/s13024-023-00640-5 37434208 PMC10334598

[B93] Jang J.-H. Lee Y. J. Ha I.-H. Park H.-J. (2024). The analgesic effect of acupuncture in neuropathic pain: Regulatory mechanisms of DNA methylation in the brain. *Pain Rep.* 9:e1200. 10.1097/PR9.0000000000001200 39450409 PMC11500783

[B94] Jhoo J. H. Kim K. W. Huh Y. Lee S. B. Park J. H. Lee J. J. (2008). Prevalence of dementia and its subtypes in an elderly urban korean population: Results from the Korean longitudinal study on health and aging (KLoSHA). *Dement. Geriatric Cogn. Disord.* 26 270–276. 10.1159/000160960 18841012

[B95] Jia L. Du Y. Chu L. Zhang Z. Li F. Lyu D. (2020). Prevalence, risk factors, and management of dementia and mild cognitive impairment in adults aged 60 years or older in China: A cross-sectional study. *Lancet Public Health* 5 e661–e671. 10.1016/S2468-2667(20)30185-7 33271079

[B96] Jiang H. (2020). *Exploration of the Relationship between Cognitive Function and Brain Iron Deposition in MCI, AD, and VCI Patients.* Master’s Thesis. Suzhou: Suzhou University.

[B97] Jiang H. Shang Z. You L. Zhang J. Jiao J. Qian Y. (2023). Electroacupuncture pretreatment at Zusanli (ST36) ameliorates hepatic ischemia/reperfusion injury in mice by reducing oxidative stress via activating vagus nerve-dependent Nrf2 pathway. *J. Inflammation Res.* 16 1595–1610. 10.2147/JIR.S404087 37092126 PMC10120822

[B98] Jiang X. Stockwell B. R. Conrad M. (2021). Ferroptosis: Mechanisms, biology and role in disease. *Nat. Rev. Mol. Cell Biol.* 22 266–282. 10.1038/s41580-020-00324-8 33495651 PMC8142022

[B99] Juarez D. Fruman D. A. (2021). Targeting the mevalonate pathway in cancer. *Trends Cancer* 7 525–540. 10.1016/j.trecan.2020.11.008 33358111 PMC8137523

[B100] Justicia C. Ramos-Cabrer P. Hoehn M. (2008). MRI detection of secondary damage after stroke: Chronic iron accumulation in the thalamus of the rat brain. *Stroke* 39 1541–1547. 10.1161/STROKEAHA.107.503565 18323485

[B101] Karatas H. Eun Jung J. Lo E. H. van Leyen K. (2018). Inhibiting 12/15-lipoxygenase to treat acute stroke in permanent and tPA induced thrombolysis models. *Brain Res.* 1678 123–128. 10.1016/j.brainres.2017.10.024 29079502 PMC5714685

[B102] Kenny E. M. Fidan E. Yang Q. Anthonymuthu T. S. New L. A. Meyer E. A. (2019). Ferroptosis contributes to neuronal death and functional outcome after traumatic brain injury. *Crit. Care Med.* 47 410–418. 10.1097/CCM.0000000000003555 30531185 PMC6449247

[B103] Kim M.-W. Chung Y. C. Jung H. C. Park M.-S. Han Y.-M. Chung Y.-A. (2012). Electroacupuncture enhances motor recovery performance with brain-derived neurotrophic factor expression in rats with cerebral infarction. *Acupuncture Med.* 30 222–226. 10.1136/acupmed-2011-010126 22729070

[B104] Kim S.-W. Kim Y. Kim S. E. An J.-Y. (2021). Ferroptosis-related genes in neurodevelopment and central nervous system. *Biology* 10:35. 10.3390/biology10010035 33419148 PMC7825574

[B105] Kirsch W. McAuley G. Holshouser B. Petersen F. Ayaz M. Vinters H. V. (2009). Serial susceptibility weighted MRI measures brain iron and microbleeds in dementia. *J. Alzheimer’s Dis.* 17 599–609. 10.3233/JAD-2009-1073 19433895 PMC2788087

[B106] Kitsugi K. Noritake H. Matsumoto M. Hanaoka T. Umemura M. Yamashita M. (2023). Simvastatin inhibits hepatic stellate cells activation by regulating the ferroptosis signaling pathway. *Biochim. Biophys. Acta Mol. Basis Dis.* 1869:166750. 10.1016/j.bbadis.2023.166750 37268254

[B107] Kondo Y. Ogawa N. Asanuma M. Ota Z. Mori A. (1995). Regional differences in late-onset iron deposition, ferritin, transferrin, astrocyte proliferation, and microglial activation after transient forebrain ischemia in rat brain. *J. Cereb. Blood Flow Metab.* 15 216–226. 10.1038/jcbfm.1995.27 7860655

[B108] Kuchcinski G. Munsch F. Lopes R. Bigourdan A. Su J. Sagnier S. (2017). Thalamic alterations remote to infarct appear as focal iron accumulation and impact clinical outcome. *Brain J. Neurol.* 140 1932–1946. 10.1093/brain/awx114 28549087 PMC6248443

[B109] Kuhn H. Banthiya S. van Leyen K. (2015). Mammalian lipoxygenases and their biological relevance. *Biochim. Biophys. Acta* 1851 308–330. 10.1016/j.bbalip.2014.10.002 25316652 PMC4370320

[B110] Lang J. Luo J. Wang L. Xu W. Jia J. Zhao Z. (2024). Electroacupuncture suppresses oxidative stress and ferroptosis by activating the mTOR/SREBP1 pathway in ischemic stroke. *Crit. Rev. Immunol.* 44 99–110. 10.1615/CritRevImmunol.2024051934 38848297

[B111] Lee Y.-C. Li T.-M. Tzeng C.-Y. Chen Y.-I. Ho W.-J. Lin J.-G. (2011). Electroacupuncture at the Zusanli (ST-36) acupoint induces a hypoglycemic effect by stimulating the cholinergic nerve in a rat model of streptozotocine-induced insulin-dependent diabetes mellitus. *Evid. Based Compl. Alternative Med.* 2011:650263. 10.1093/ecam/neq068 21799686 PMC3136799

[B112] Lee Y.-M. Choi D.-H. Cheon M.-W. Kim J. G. Kim J.-S. Shin M.-G. (2022). Changes in mitochondria-related gene expression upon acupuncture at LR3 in the D-galactosamine-induced liver damage rat model. *Evid. Based Compl. Alternative Med.* 2022:3294273. 10.1155/2022/3294273 35928244 PMC9345726

[B113] Levi S. Ripamonti M. Moro A. S. Cozzi A. (2024). Iron imbalance in neurodegeneration. *Mol. Psychiatry* 29 1139–1152. 10.1038/s41380-023-02399-z 38212377 PMC11176077

[B114] Li H. Choi H. Houser M. C. Li C. Liu T. Gao S. (2024). Impact of acupuncture on human metabolomic profiles: A systematic review. *Metabolites* 14:542. 10.3390/metabo14100542 39452923 PMC11509109

[B115] Li J. He Y. Du Y.-H. Zhang M. Georgi R. Kolberg B. (2022). Effect of electro-acupuncture on vasomotor symptoms in rats with acute cerebral infarction based on phosphatidylinositol system. *Chin. J. Integr. Med.* 28 145–152. 10.1007/s11655-021-3341-6 34874522 PMC8649319

[B116] Li M. Dai X. Kuang B. Yu X. Teng W. Yu W. (2021). The effect of acupuncture on iron induced cell death in brain tissue of rats with cerebral hemorrhage. *J. Tradit. Chin. Med.* 49 61–67. 10.19664/j.cnki.1002-2392.210264

[B117] Li M.-Y. Dai X.-H. Yu X.-P. Zou W. Teng W. Liu P. (2022). Scalp acupuncture protects against neuronal ferroptosis by activating the p62-Keap1-Nrf2 pathway in rat models of intracranial haemorrhage. *J. Mol. Neurosci.* 72 82–96. 10.1007/s12031-021-01890-y 34405366 PMC8755669

[B118] Li N. Wang H. Liu H. Zhu L. Lyu Z. Qiu J. (2023). The effects and mechanisms of acupuncture for post-stroke cognitive impairment: Progress and prospects. *Front. Neurosci.* 17:1211044. 10.3389/fnins.2023.1211044 37397457 PMC10309044

[B119] Li X. Jin D. Zhu Y. Liu L. Qiao Y. Qian Y. (2021). Quantitative susceptibility mapping to evaluate brain iron deposition and its correlation with physiological parameters in hypertensive patients. *Ann. Transl. Med.* 9:1582. 10.21037/atm-21-5170 34790788 PMC8576670

[B120] Li Y. Hao Y. Yang Y. Lv Z. (2018). Research progress on ferroptosis in neurological diseases. *J. Apooplexy Nervous Dis.* 35 762–765. 10.19845/j.cnki.zfysjjbzz.2018.08.017

[B121] Li Y. He Y. Guan Q. Liu W. Han H. Nie Z. (2012). Disrupted iron metabolism and ensuing oxidative stress may mediate cognitive dysfunction induced by chronic cerebral hypoperfusion. *Biol. Trace Element Res.* 150 242–248. 10.1007/s12011-012-9455-0 22639386

[B122] Li Y. Ran Q. Duan Q. Jin J. Wang Y. Yu L. (2024). 7-Dehydrocholesterol dictates ferroptosis sensitivity. *Nature* 626 411–418. 10.1038/s41586-023-06983-9 38297130 PMC11298758

[B123] Li Y. Xiao D. Wang X. (2022). The emerging roles of ferroptosis in cells of the central nervous system. *Front. Neurosci.* 16:1032140. 10.3389/fnins.2022.1032140 36590286 PMC9797129

[B124] Liang D. Minikes A. M. Jiang X. (2022). Ferroptosis at the intersection of lipid metabolism and cellular signaling. *Mol. Cell.* 82 2215–2227. 10.1016/j.molcel.2022.03.022 35390277 PMC9233073

[B125] Liang R. (2023). *Study on the Mechanism of Electroacupuncture Regulating Brain Iron Metabolism and Reducing Oxidative Damage After Cerebral Ischemia-Reperfusion Injury.* Master’s thesis. Heilongjiang: Heilongjiang University Of Chinese Medicine, 10.27127/d.cnki.ghlzu.2022.000441

[B126] Liang R. Tang Q. Song W. Zhang M. Teng L. Kang Y. (2021). Electroacupuncture preconditioning reduces oxidative stress in the acute phase of cerebral ischemia-reperfusion in rats by regulating iron metabolism pathways. *Evid. Based Compl. Alternative Med.* 2021:3056963. 10.1155/2021/3056963 34790244 PMC8592755

[B127] Lin B. Wang M. Chen X. Chai L. Ni J. Huang J. (2024). Involvement of P2X7R-mediated microglia polarization and neuroinflammation in the response to electroacupuncture on post-stroke memory impairment. *Brain Res. Bull.* 212:110967. 10.1016/j.brainresbull.2024.110967 38670470

[B128] Lin R. Lin Y. Tao J. Chen B. Yu K. Chen J. (2015). Electroacupuncture ameliorates learning and memory in rats with cerebral ischemia-reperfusion injury by inhibiting oxidative stress and promoting p-CREB expression in the hippocampus. *Mol. Med. Rep.* 12 6807–6814. 10.3892/mmr.2015.4321 26397995

[B129] Lin R. Yu K. Li X. Tao J. Lin Y. Zhao C. (2016). Electroacupuncture ameliorates post-stroke learning and memory through minimizing ultrastructural brain damage and inhibiting the expression of MMP-2 and MMP-9 in cerebral ischemia-reperfusion injured rats. *Mol. Med. Rep.* 14 225–233. 10.3892/mmr.2016.5227 27177163 PMC4918523

[B130] Liu C. Li C. Yang J. Gui L. Zhao L. Evans A. C. (2015). Characterizing brain iron deposition in subcortical ischemic vascular dementia using susceptibility-weighted imaging: An in vivo MR study. *Behav. Brain Res.* 288 33–38. 10.1016/j.bbr.2015.04.003 25862942

[B131] Liu C.-Z. Yu J.-C. Zhang X.-Z. Fu W.-W. Wang T. Han J.-X. (2006). Acupuncture prevents cognitive deficits and oxidative stress in cerebral multi-infarction rats. *Neurosci. Lett.* 393 45–50. 10.1016/j.neulet.2005.09.049 16236447

[B132] Liu J. Hu X. Xue Y. Liu C. Liu D. Shang Y. (2020). Targeting hepcidin improves cognitive impairment and reduces iron deposition in a diabetic rat model. *Am. J. Transl. Res.* 12 4830–4839. 32913554 PMC7476126

[B133] Liu P. Zhang Z. Cai Y. Li Z. Zhou Q. Chen Q. (2024). Ferroptosis: Mechanisms and role in diabetes mellitus and its complications. *Ageing Res. Rev.* 94:102201. 10.1016/j.arr.2024.102201 38242213

[B134] Liu S. Gao X. Zhou S. (2022). New target for prevention and treatment of neuroinflammation: Microglia iron accumulation and ferroptosis. *ASN Neuro* 14:17590914221133236. 10.1177/17590914221133236 36285433 PMC9607999

[B135] Liu X. Zhang L. Zhang H. Lai L. Wang Y. Sun J. (2022). Low-frequency electroacupuncture improves disordered hepatic energy metabolism in insulin-resistant Zucker diabetic fatty rats via the AMPK/mTORC1/p70S6K signaling pathway. *Acupuncture Med*. 40 360–368. 10.1177/09645284211070301 35034504

[B136] Liu X.-Y. Dai X.-H. Zou W. Yu X.-P. Teng W. Wang Y. (2018). Acupuncture through Baihui (DU20) to Qubin (GB7) mitigates neurological impairment after intracerebral hemorrhage. *Neural Regeneration Res.* 13 1425–1432. 10.4103/1673-5374.235298 30106055 PMC6108213

[B137] Liu Y. Wan Y. Jiang Y. Zhang L. Cheng W. (2023). GPX4: The hub of lipid oxidation, ferroptosis, disease and treatment. *Biochim. Biophys. Acta Rev. Cancer* 1878:188890. 10.1016/j.bbcan.2023.188890 37001616

[B138] Long H. Zhu W. Wei L. Zhao J. (2023). Iron homeostasis imbalance and ferroptosis in brain diseases. *MedComm* 4:e298. 10.1002/mco2.298 37377861 PMC10292684

[B139] Lüders S. Stöve S. Schrader J. (2012). [Prevention of vascular dementia. evidence and practice]. *Der. Internist* 53 223–231. 10.1007/s00108-011-2953-x 22119909

[B140] Luo A. Xie Z. Wang Y. Wang X. Li S. Yan J. (2022). Type 2 diabetes mellitus-associated cognitive dysfunction: Advances in potential mechanisms and therapies. *Neurosci. Biobehav. Rev.* 137:104642. 10.1016/j.neubiorev.2022.104642 35367221

[B141] Luo E.-F. Li H.-X. Qin Y.-H. Qiao Y. Yan G.-L. Yao Y.-Y. (2021). Role of ferroptosis in the process of diabetes-induced endothelial dysfunction. *World J. Diabetes* 12 124–137. 10.4239/wjd.v12.i2.124 33594332 PMC7839168

[B142] Luo J. Yang H. Song B.-L. (2020). Mechanisms and regulation of cholesterol homeostasis. *Nat. Rev. Mol. Cell Biol.* 21 225–245. 10.1038/s41580-019-0190-7 31848472

[B143] Luobin D. Huajun W. Yao L. I. Jia L. I. Ling L. I. Yangping G. (2023). Electroacupuncture stimulating Neixiyan (EX-LE5) and Dubi (ST35) alleviates osteoarthritis in rats induced by anterior cruciate ligament transaction affecting DNA methylation regulated transcription of miR-146a and miR-140-5p. *J. Traditional Chin. Med.* 43 983–990. 10.19852/j.cnki.jtcm.2023.05.004 37679986 PMC10465835

[B144] Ma Z. (2023). *Study on the Effect of Acupuncture Treatment on Mild Vascular Cognitive Impairment Based on Plasma Metabolomics.* Dissertation/master’s thesis. Tianjin: University of Traditional Chinese Medicine, 10.27368/d.cnki.gtzyy.2022.000503

[B145] Madden D. J. Merenstein J. L. (2023). Quantitative susceptibility mapping of brain iron in healthy aging and cognition. *NeuroImage* 282:120401. 10.1016/j.neuroimage.2023.120401 37802405 PMC10797559

[B146] Majerníková N. den Dunnen W. F. A. Dolga A. M. (2021). The potential of ferroptosis-targeting therapies for Alzheimer’s disease: From mechanism to transcriptomic analysis. *Front. Aging Neurosci.* 13:745046. 10.3389/fnagi.2021.745046 34987375 PMC8721139

[B147] Majerníková N. Marmolejo-Garza A. Salinas C. S. Luu M. D. A. Zhang Y. Trombetta-Lima M. (2024). The link between amyloid β and ferroptosis pathway in Alzheimer’s disease progression. *Cell Death Dis.* 15:782. 10.1038/s41419-024-07152-0 39468028 PMC11519607

[B148] Matsumoto L. Suzuki K. Mizuno Y. Ohike Y. Ozeki A. Ono S. (2018). Association of subclinical carotid atherosclerosis with immediate memory and other cognitive functions. *Geriatrics Gerontol. Int.* 18 65–71. 10.1111/ggi.13142 28776906

[B149] McNeill A. Birchall D. Hayflick S. J. Gregory A. Schenk J. F. Zimmerman E. A. (2008). T2* and FSE MRI distinguishes four subtypes of neurodegeneration with brain iron accumulation. *Neurology* 70 1614–1619. 10.1212/01.wnl.0000310985.40011.d6 18443312 PMC2706154

[B150] Mehta S. H. Webb R. C. Ergul A. Tawfik A. Dorrance A. M. (2004). Neuroprotection by tempol in a model of iron-induced oxidative stress in acute ischemic stroke. *Am. J. Physiol. Regulatory Integr. Comp. Physiol.* 286 R283–R288. 10.1152/ajpregu.00446.2002 14592931

[B151] Mendes A. Herrmann F. R. Scheffler M. Gabriel G. Sveikata L. Rakotomiaramanana B. (2020). Cortical superficial siderosis: A descriptive analysis in a memory clinic population. *J. Alzheimer’s Dis.* 73 1467–1479. 10.3233/JAD-190619 31929155

[B152] Milenkovic D. Nuthikattu S. Norman J. E. Villablanca A. C. (2024). Global genomic profile of hippocampal endothelial cells by single-nuclei RNA sequencing in female diabetic mice is associated with cognitive dysfunction. *Am. J. Physiol. Heart Circulatory Physiol.* 327 H908–H926. 10.1152/ajpheart.00251.2024 39150395 PMC11901383

[B153] Millerot-Serrurot E. Bertrand N. Mossiat C. Faure P. Prigent-Tessier A. Garnier P. (2008). Temporal changes in free iron levels after brain ischemia Relevance to the timing of iron chelation therapy in stroke. *Neurochem. Int.* 52 1442–1448. 10.1016/j.neuint.2008.04.002 18485533

[B154] Ming M. Hu W. Xie G. Chen J. Huang Y. (2023). Dendrobium nobile polysaccharides attenuates ferroptosis and improves cognitive function in vascular dementia rats. *Am. J. Alzheimer’s Dis. Other Dement.* 38:15333175231185236. 10.1177/15333175231185236 37342000 PMC10623970

[B155] Moon Y. Han S.-H. Moon W.-J. (2016). Patterns of brain iron accumulation in vascular dementia and Alzheimer’s dementia using quantitative susceptibility mapping imaging. *J. Alzheimer’s Dis.* 51 737–745. 10.3233/JAD-151037 26890777

[B156] Mortensen M. S. Ruiz J. Watts J. L. (2023). Polyunsaturated fatty acids drive lipid peroxidation during ferroptosis. *Cells* 12:804. 10.3390/cells12050804 36899940 PMC10001165

[B157] Mou Y. Wang J. Wu J. He D. Zhang C. Duan C. (2019). Ferroptosis, a new form of cell death: Opportunities and challenges in cancer. *J. Hematol. Oncol.* 12:34. 10.1186/s13045-019-0720-y 30925886 PMC6441206

[B158] Ni H. Ren J. Wang Q. Li X. Wu Y. Liu D. (2023). Electroacupuncture at ST 36 ameliorates cognitive impairment and beta-amyloid pathology by inhibiting NLRP3 inflammasome activation in an Alzheimer’s disease animal model. *Heliyon* 9:e16755. 10.1016/j.heliyon.2023.e16755 37292305 PMC10245255

[B159] Ogawa T. Yoshida Y. Okudera T. Noguchi K. Kado H. Uemura K. (1997). Secondary thalamic degeneration after cerebral infarction in the middle cerebral artery distribution: Evaluation with MR imaging. *Radiology* 204 255–262. 10.1148/radiology.204.1.9205256 9205256

[B160] Ou M. Jiang Y. Ji Y. Zhou Q. Du Z. Zhu H. (2022). Role and mechanism of ferroptosis in neurological diseases. *Mol. Metab.* 61:101502. 10.1016/j.molmet.2022.101502 35447365 PMC9170779

[B161] Pan Y. Liang J. Zhang W. Gao D. Li C. Xie W. (2024). Association between age at diagnosis of hyperlipidemia and subsequent risk of dementia. *J. Am. Med. Dir. Assoc.* 25:104960. 10.1016/j.jamda.2024.01.029 38453136

[B162] Park M. W. Cha H. W. Kim J. Kim J. H. Yang H. Yoon S. (2021). NOX4 promotes ferroptosis of astrocytes by oxidative stress-induced lipid peroxidation via the impairment of mitochondrial metabolism in Alzheimer’s diseases. *Redox Biol.* 41:101947. 10.1016/j.redox.2021.101947 33774476 PMC8027773

[B163] Pendlebury S. T. Rothwell P. M. (2009). Prevalence, incidence, and factors associated with pre-stroke and post-stroke dementia: A systematic review and meta-analysis. *Lancet Neurol.* 8 1006–1018. 10.1016/S1474-4422(09)70236-4 19782001

[B164] Peng Y. Wang C. Xu H. H. Liu Y.-N. Zhou F. (2010). Binding of alpha-synuclein with Fe(III) and with Fe(II) and biological implications of the resultant complexes. *J. Inorganic Biochem.* 104 365–370. 10.1016/j.jinorgbio.2009.11.005 20005574 PMC2824027

[B165] Perez M. A. Magtanong L. Dixon S. J. Watts J. L. (2020). Dietary lipids induce ferroptosis in caenorhabditiselegans and human cancer cells. *Developmental Cell* 54 447–454.e4. 10.1016/j.devcel.2020.06.019 32652074 PMC7483868

[B166] Pirillo A. Norata G. D. Catapano A. L. (2013). LOX-1, OxLDL, and atherosclerosis. *Mediators Inflammation* 2013:152786. 10.1155/2013/152786 23935243 PMC3723318

[B167] Plascencia-Villa G. Perry G. (2021). Preventive and therapeutic strategies in Alzheimer’s disease: Focus on oxidative stress, redox metals, and ferroptosis. *Antioxidants Redox Signaling* 34 591–610. 10.1089/ars.2020.8134 32486897 PMC8098758

[B168] Poltorack C. D. Dixon S. J. (2022). Understanding the role of cysteine in ferroptosis: Progress & paradoxes. *FEBS J.* 289 374–385. 10.1111/febs.15842 33773039 PMC8473584

[B169] Pope L. E. Dixon S. J. (2023). Regulation of ferroptosis by lipid metabolism. *Trends Cell Biol.* 33 1077–1087. 10.1016/j.tcb.2023.05.003 37407304 PMC10733748

[B170] Pozojevic J. Spielmann M. (2023). Single-cell sequencing in neurodegenerative disorders. *Mol. Diagnosis Therapy* 27 553–561. 10.1007/s40291-023-00668-9 37552451 PMC10435411

[B171] Prajjwal P. Marsool M. D. M. Inban P. Sharma B. Asharaf S. Aleti S. (2023). Vascular dementia subtypes, pathophysiology, genetics, neuroimaging, biomarkers, and treatment updates along with its association with Alzheimer’s dementia and diabetes mellitus. *Disease-a-Month* 69:101557. 10.1016/j.disamonth.2023.101557 37031059

[B172] Qin W. Zhou Z. Lu J. (2023). Research progress on the mechanism of ferroptosis and its intervention in ischemic stroke. *J. Integr. Cardio. Cerebrovasc. Dis.* 21 4558–4564. 10.12114/j.issn.1008-5971.2022.00.185

[B173] Qin Z. Wu W. Liu D. Zheng C. Kang J. Zhou H. (2022). Quantitative susceptibility mapping of brain iron relating to cognitive impairment in hypertension. *J. Magn. Reson. Imaging* 56 508–515. 10.1002/jmri.28043 34989062

[B174] Qiu B. Zandkarimi F. Bezjian C. T. Reznik E. Soni R. K. Gu W. (2024). Phospholipids with two polyunsaturated fatty acyl tails promote ferroptosis. *Cell* 187 1177–1190.e18. 10.1016/j.cell.2024.01.030 38366593 PMC10940216

[B175] Ratan R. R. (2020). The chemical biology of ferroptosis in the central nervous system. *Cell Chem. Biol.* 27 479–498. 10.1016/j.chembiol.2020.03.007 32243811 PMC7245561

[B176] Reichert C. O. de Freitas F. A. Sampaio-Silva J. Rokita-Rosa L. Barros P. de L. (2020). Ferroptosis mechanisms involved in neurodegenerative diseases. *Int. J. Mol. Sci.* 21:22. 10.3390/ijms21228765 33233496 PMC7699575

[B177] Reitz C. Tang M.-X. Luchsinger J. Mayeux R. (2004). Relation of plasma lipids to Alzheimer disease and vascular dementia. *Arch. Neurol.* 61 705–714. 10.1001/archneur.61.5.705 15148148 PMC2696387

[B178] Ru Q. Li Y. Chen L. Wu Y. Min J. Wang F. (2024). Iron homeostasis and ferroptosis in human diseases: Mechanisms and therapeutic prospects. *Signal Transduction Targeted Therapy* 9 1–64. 10.1038/s41392-024-01969-z 39396974 PMC11486532

[B179] Rundek T. Tolea M. Ariko T. Fagerli E. A. Camargo C. J. (2022). Vascular cognitive impairment (VCI). *Neurother. J. Am. Soc. Exp. NeuroTherapeutics* 19 68–88. 10.1007/s13311-021-01170-y 34939171 PMC9130444

[B180] Ryan F. Blex C. Ngo T. D. Kopp M. A. Michalke B. Venkataramani V. (2024). Ferroptosis inhibitor improves outcome after early and delayed treatment in mild spinal cord injury. *Acta Neuropathol.* 147:106. 10.1007/s00401-024-02758-2 38907771 PMC11193702

[B181] Seibt T. M. Proneth B. Conrad M. (2019). Role of GPX4 in ferroptosis and its pharmacological implication. *Free Radical Biol. Med.* 133 144–152. 10.1016/j.freeradbiomed.2018.09.014 30219704

[B182] Sethi S. K. Sharma S. Gharabaghi S. Reese D. Chen Y. Adams P. (2022). Quantifying brain iron in hereditary hemochromatosis using R2* and susceptibility mapping. *AJNR Am. J. Neuroradiol.* 43 991–997. 10.3174/ajnr.A7560 35798390 PMC9262054

[B183] Sha W. Hu F. Xi Y. Chu Y. Bu S. (2021). Mechanism of ferroptosis and its role in type 2 diabetes mellitus. *J. Diabetes Res.* 2021:9999612. 10.1155/2021/9999612 34258295 PMC8257355

[B184] Shah R. Shchepinov M. S. Pratt D. A. (2018). Resolving the role of lipoxygenases in the initiation and execution of ferroptosis. *ACS Central Sci.* 4 387–396. 10.1021/acscentsci.7b00589 29632885 PMC5879472

[B185] Shang G. Shao Q. Lv K. Xu W. Ji J. Fan S. (2024). Hypercholesterolemia and the increased risk of vascular dementia: A cholesterol perspective. *Curr. Atherosc. Rep.* 26 435–449. 10.1007/s11883-024-01217-3 38814418

[B186] Sharrett A. R. Patsch W. Sorlie P. D. Heiss G. Bond M. G. Davis C. E. (1994). Associations of lipoprotein cholesterols, apolipoproteins A-I and B, and triglycerides with carotid atherosclerosis and coronary heart disease. The Atherosclerosis Risk in Communities (ARIC) study. *Arteriosc. Thrombosis J. Vascular Biol.* 14 1098–1104. 10.1161/01.atv.14.7.1098 8018665

[B187] Shen Z.-Q. Chang W.-Q. Liang L.-F. Zhang J.-R. Wang Y.-Q. Yin X. (n.d.). *Electroacupuncture Effects on Trigeminal Neuralgia with Comorbid Anxiety and Depression: The Role of Frequency and Acupoint Specificity.* 10.1096/fj.202402461RR 39840659

[B188] Skrobot O. A. O’Brien J. Black S. Chen C. DeCarli C. Erkinjuntti T. (2017). The vascular impairment of cognition classification consensus study, Alzheimer’s Dement. *J. Alzheimer’s Assoc.* 13 624–633. 10.1016/j.jalz.2016.10.007 27960092

[B189] Sotoudeh H. Sarrami A. H. Wang J.-X. Saadatpour Z. Razaei A. Gaddamanugu S. (2021). Susceptibility-weighted imaging in neurodegenerative disorders: A review. *J. Neuroimaging* 31 459–470. 10.1111/jon.12841 33624404

[B190] Spence H. McNeil C. J. Waiter G. D. (2022). Cognition and brain iron deposition in whole grey matter regions and hippocampal subfields. *Eur. J. Neurosci.* 56 6039–6054. 10.1111/ejn.15838 36215153 PMC10092357

[B191] Stockwell B. R. (2022). Ferroptosis turns 10: Emerging mechanisms, physiological functions, and therapeutic applications. *Cell* 185 2401–2421. 10.1016/j.cell.2022.06.003 35803244 PMC9273022

[B192] Su X.-T. Wang L. Ma S.-M. Cao Y. Yang N.-N. Lin L.-L. (2020). Mechanisms of acupuncture in the regulation of oxidative stress in treating ischemic stroke. *Oxid. Medi. Cell. Longevity* 2020:7875396. 10.1155/2020/7875396 33178387 PMC7644298

[B193] Suda A. Umaru B. A. Yamamoto Y. Shima H. Saiki Y. Pan Y. (2024). Polyunsaturated fatty acids-induced ferroptosis suppresses pancreatic cancer growth. *Sci. Rep.* 14:4409. 10.1038/s41598-024-55050-4 38388563 PMC10884029

[B194] Sun P. Chu H. Li N. Liu H. Liu S. Zhang L. (2022). The effect of “Tongdu Tiaoshen” acupuncture on the CREB/BDNF/TrkB signaling pathway in the hippocampus of post-stroke depression rats. *Zhongguo Zhen Jiu* 42 907–913. 10.13703/j.0255-2930.20220206-k0003 35938334

[B195] Sun Q. Liu D. Cui W. Cheng H. Huang L. Zhang R. (2023). Cholesterol mediated ferroptosis suppression reveals essential roles of Coenzyme Q and squalene. *Commun. Biol.* 6:1108. 10.1038/s42003-023-05477-8 37914914 PMC10620397

[B196] Sun Y. Ge X. Han X. Cao W. Wang Y. Ding W. (2017). Characterizing brain iron deposition in patients with subcortical vascular mild cognitive impairment using quantitative susceptibility mapping: A potential biomarker. *Front. Aging Neurosci.* 9:81. 10.3389/fnagi.2017.00081 28424610 PMC5371674

[B197] Tang D. Kroemer G. (2020). Peroxisome: The new player in ferroptosis. *Signal Transduction Targeted Therapy* 5 273. 10.1038/s41392-020-00404-3 33235217 PMC7686316

[B198] Tang D. Chen X. Kang R. Kroemer G. (2021). Ferroptosis: Molecular mechanisms and health implications. *Cell Res.* 31 107–125. 10.1038/s41422-020-00441-1 33268902 PMC8026611

[B199] Tang D. Kang R. Berghe T. V. Vandenabeele P. Kroemer G. (2019). The molecular machinery of regulated cell death. *Cell Res.* 29 347–364. 10.1038/s41422-019-0164-5 30948788 PMC6796845

[B200] Tang W. Qin J. Zhou Y. Wang W. Teng F. Liu J. (2023). Regulation of ferroptosis and ACSL4-15LO1 pathway contributed to the anti-asthma effect of acupuncture. *Int. Immunopharmacol.* 115:109670. 10.1016/j.intimp.2022.109670 36603356

[B201] Tanner L. I. Lienhard G. E. (1987). Insulin elicits a redistribution of transferrin receptors in 3T3-L1 adipocytes through an increase in the rate constant for receptor externalization. *J. Biol. Chem.* 262 8975–8980. 3298247

[B202] Tao J. Zheng Y. Liu W. Yang S. Huang J. Xue X. (2016). Electro-acupuncture at LI11 and ST36 acupoints exerts neuroprotective effects via reactive astrocyte proliferation after ischemia and reperfusion injury in rats. *Brain Res. Bull.* 120 14–24. 10.1016/j.brainresbull.2015.10.011 26524137

[B203] Tian X. Li X. Pan M. Yang L. Z. Li Y. Fang W. (2024). Study on the effect of acupuncture treatment on mild vascular cognitive impairment based on plasma metabolomics. *Cell. Mol. Neurobiol.* 44:25. 10.1007/s10571-024-01457-6 38393376 PMC10891262

[B204] Tu C.-H. MacDonald I. Chen Y.-H. (2019). The effects of acupuncture on glutamatergic neurotransmission in depression, anxiety, schizophrenia, and Alzheimer’s disease: A review of the literature. *Front. Psychiatry* 10:14. 10.3389/fpsyt.2019.00014 30809158 PMC6379324

[B205] Tuo Q.-Z. Lei P. Jackman K. A. Li X.-L. Xiong H. Li X.-L. (2017). Tau-mediated iron export prevents ferroptotic damage after ischemic stroke. *Mol. Psychiatry* 22 1520–1530. 10.1038/mp.2017.171 28886009

[B206] Ursini F. Maiorino M. (2020). Lipid peroxidation and ferroptosis: The role of GSH and GPx4. *Free Radical Biol. Med.* 152 175–185. 10.1016/j.freeradbiomed.2020.02.027 32165281

[B207] van Etten E. S. van der Grond J. Dumas E. M. van den Bogaard S. J. A. van Buchem M. A. Wermer M. J. H. (2015). MRI susceptibility changes suggestive of iron deposition in the thalamus after ischemic stroke. *Cerebrovasc. Dis.* 40 67–72. 10.1159/000433560 26184716

[B208] Velasquez J. Wray A. A. (2023). “Deferoxamine,” in *StatPearls [Internet]*. eds. StatPearls Editorial Team (Treasure Island, FL: StatPearls Publishing).

[B209] Vinayagamani S. Sheelakumari R. Sabarish S. Senthilvelan S. Ros R. Thomas B. (2021). Quantitative susceptibility mapping: Technical considerations and clinical applications in neuroimaging. *J. Magn. Reson. Imaging* 53 23–37. 10.1002/jmri.27058 31951057

[B210] Wan K. Jia M. Zhang H. Lan Y. Wang S. Zhang K. (2023). Electroacupuncture alleviates neuropathic pain by suppressing ferroptosis in dorsal root ganglion via SAT1/ALOX15 signaling. *Mol. Neurobiol.* 60 6121–6132. 10.1007/s12035-023-03463-z 37421564

[B211] Wanders R. J. A. Baes M. Ribeiro D. Ferdinandusse S. Waterham H. R. (2023). The physiological functions of human peroxisomes. *Physiol. Rev.* 103 957–1024. 10.1152/physrev.00051.2021 35951481

[B212] Wang G.-L. Xu S.-Y. Lv H.-Q. Zhang C. Peng Y.-J. (2023). Electroacupuncture inhibits ferroptosis induced by cerebral ischemiareperfusion. *Curr. Neurovasc. Res.* 20 346–353. 10.2174/1567202620666230623153728 37357521

[B213] Wang H. Tang H. Jiang S. Li Z. Lv Q. Tian H. (2022). The effect of acupuncture on ferroptosis in hippocampal tissue of rats with cerebral ischemia-reperfusion injury. *J. Hunan. Univ. Chin. Med.* 42 1683–1687.

[B214] Wang H. Xu L. Tang X. Jiang Z. Feng X. (2024). Lipid peroxidation-induced ferroptosis as a therapeutic target for mitigating neuronal injury and inflammation in sepsis-associated encephalopathy: Insights into the hippocampal PEBP-1/15-LOX/GPX4 pathway. *Lipids Health Dis.* 23:128. 10.1186/s12944-024-02116-x 38685023 PMC11057122

[B215] Wang L. (2023). *Effect of Electroacupuncture on Lipid Metabolism Pathway of Ferroptosis in Myocardial.* Master’s thesis. Hefei: Anhui University of Chinese Medicine, 10.26922/d.cnki.ganzc.2022.000284

[B216] Wang L. Liu T. Guo J. Zhao T. Tang H. Dong F. (2023). Sex differences in erythrocyte fatty acid composition of first-diagnosed, drug-naïve patients with major depressive disorders. *Front. Pharmacol.* 14:1314151. 10.3389/fphar.2023.1314151 38164472 PMC10757913

[B217] Wang L. Yang J.-W. Lin L.-T. Huang J. Wang X.-R. Su X.-T. (n.d.). *Acupuncture Attenuates Inflammation in Microglia of Vascular Dementia Rats by Inhibiting miR-93-Mediated TLR4/MyD88/NF-κB Signaling Pathway.* 10.1155/2020/8253904 32850002 PMC7441436

[B218] Wang Q. Li P. Xu J. (2024). Analysis of the protective mechanism of acupuncture on the brain of rats with cerebral ischemia-reperfusion injury from the perspective of iron death pathway. *Liaoning J. Tradit. Chin. Med.* 51 196–200. 10.13192/j.issn.1000-1719.2024.06.052

[B219] Wang Y. Wu S. Li Q. Sun H. Wang H. (2023). Pharmacological inhibition of ferroptosis as a therapeutic target for neurodegenerative diseases and strokes. *Adv. Sci.* 10:2300325. 10.1002/advs.202300325 37341302 PMC10460905

[B220] Weiland A. Wang Y. Wu W. Lan X. Han X. Li Q. (2019). Ferroptosis and its role in diverse brain diseases. *Mol. Neurobiol.* 56 4880–4893. 10.1007/s12035-018-1403-3 30406908 PMC6506411

[B221] Wu J.-R. Tuo Q.-Z. Lei P. (2018). Ferroptosis, a recent defined form of critical cell death in neurological disorders. *J. Mol. Neurosci.* 66 197–206. 10.1007/s12031-018-1155-6 30145632

[B222] Wu R. Wu L. Qi S. Gao J. Ding Z. (2024). Study on the mechanism of “Shugan Tiaoshen” acupuncture regulating lipidperoxidation stress inhibiting iron death of prefrontal neurons and improving post-stroke depression. *Acupunct. Res.* doi: 11.2274.R.20240712.1420.002 Advance online publication.

[B223] Wu X. Wang Y. Han W. Zhang L. Zhang J. Zhang G. (2023). The effect of electroacupuncture pretreatment on neuronal ferroptosis in rats with cerebral ischemia-reperfusion injury. *Zhen Ci Yan Jiu* 48 754–763. 10.13702/j.1000-0607.20230148 37614133

[B224] Wu Z.-F. Peng H.-H. Shu Y. Zhang L. Zhang S. Zhang J.-Y. (2024). Electroacupuncture activates the peroxisome proliferators-activated receptor pathway to improve the phenotype of cerebral palsy. *CNS Neurosci. Therapeutics* 30:e14876. 10.1111/cns.14876 39049731 PMC11269887

[B225] Xia W.-G. Zheng C.-J. Zhang X. Wang J. (2017). Effects of “nourishing liver and kidney” acupuncture therapy on expression of brain derived neurotrophic factor and synaptophysin after cerebral ischemia reperfusion in rats. *J. Huazhong Univer. Sci. Technol. Med. Sci.* 37 271–278. 10.1007/s11596-017-1727-7 28397041

[B226] Xie X.-C. Cao Y.-Q. Gao Q. Wang C. Li M. Wei S.-G. (2017). Acupuncture improves intestinal absorption of iron in iron-deficient obese patients: A randomized controlled preliminary trial. *Chin. Med. J.* 130 508–515. 10.4103/0366-6999.200549 28229980 PMC5339922

[B227] Xie Z. (2023). *The Mechanism of Hippocampal Neuronal Ferroptosis Mediating Cognitive Dysfunction in Mice with Type 2 Diabetes.* Master’s thesis. Wuhan: Huazhong University of Science and Technology, 10.27157/d.cnki.ghzku.2023.004661

[B228] Yan H. Zou T. Tuo Q. Xu S. Li H. Belaidi A. A. (2021). Ferroptosis: Mechanisms and links with diseases. *Signal Transduction Targeted Therapy* 6 1–16. 10.1038/s41392-020-00428-9 33536413 PMC7858612

[B229] Yan N. Zhang J.-J. (2019). The emerging roles of ferroptosis in vascular cognitive impairment. *Front. Neurosci.* 13:811. 10.3389/fnins.2019.00811 31447633 PMC6691122

[B230] Yan N. Xu Z. Qu C. Zhang J. (2021). Dimethyl fumarate improves cognitive deficits in chronic cerebral hypoperfusion rats by alleviating inflammation, oxidative stress, and ferroptosis via NRF2/ARE/NF-κB signal pathway. *Int. Immunopharmacol.* 98:107844. 10.1016/j.intimp.2021.107844 34153667

[B231] Yang B. (2024). *Study on the Neuroprotective Mechanism of Acupuncture Therapy Regulating lipid Peroxidation and Inhibiting Ferroptosis in Rats with Cerebral Ischemia.* Master’s thesis. Harbin: Heilongjiang University Of Chinese Medicine, 10.27127/d.cnki.ghlzu.2023.000486

[B232] Yang J. Dai X. Xu H. Tang Q. Bi F. (2022a). Regulation of ferroptosis by amino acid metabolism in cancer. *Int. J. Biol. Sci.* 18 1695–1705. 10.7150/ijbs.64982 35280684 PMC8898355

[B233] Yang J. Hu S. Bian Y. Yao J. Wang D. Liu X. (2022b). Targeting cell death: Pyroptosis, ferroptosis, apoptosis and necroptosis in osteoarthritis. *Front. Cell Developmental Biol.* 9:789948. 10.3389/fcell.2021.789948 35118075 PMC8804296

[B234] Yang K. Song H. Yin D. (2021). PDSS2 inhibits the ferroptosis of vascular endothelial cells in atherosclerosis by activating Nrf2. *J. Cardiovasc. Pharmacol.* 77 767–776. 10.1097/FJC.0000000000001030 33929387 PMC8274586

[B235] Yang P. Chen H. Wang T. Li L. Su H. Li J. (2023). Electroacupuncture attenuates chronic inflammatory pain and depression comorbidity by inhibiting hippocampal neuronal apoptosis via the PI3K/Akt signaling pathway. *Neurosci. Lett.* 812:137411. 10.1016/j.neulet.2023.137411 37516346

[B236] Yang X. Jin Y. Ning R. Mao Q. Zhang P. Zhou L. (2025). Electroacupuncture attenuates ferroptosis by promoting Nrf2 nuclear translocation and activating Nrf2/SLC7A11/GPX4 pathway in ischemic stroke. *Chin. Med.* 20:4. 10.1186/s13020-024-01047-0 39755657 PMC11699709

[B237] Yang Y. Kimura-Ohba S. Thompson J. Rosenberg G. A. (2016). Rodent models of vascular cognitive impairment. *Translational Stroke Res.* 7 407–414. 10.1007/s12975-016-0486-2 27498679 PMC5016244

[B238] Yang Z. Shi J. Chen L. Fu C. Shi D. Qu H. (2022c). Role of pyroptosis and ferroptosis in the progression of atherosclerotic plaques. *Front. Cell Developmental Biol.* 10:811196. 10.3389/fcell.2022.811196 35186925 PMC8850398

[B239] Yao Z. Cai L. Zhao A. Yang L. Chen Z. Zhang Y. (2023). Electroacupuncture alleviates neuroinflammation by regulating microglia polarization via STAT6/PPARγ in ischemic stroke rats. *Neuroscience* 532 23–36. 10.1016/j.neuroscience.2023.09.007 37741355

[B240] Ye L. Wen X. Qin J. Zhang X. Wang Y. Wang Z. (2024). Metabolism-regulated ferroptosis in cancer progression and therapy. *Cell Death Dis.* 15 1–12. 10.1038/s41419-024-06584-y 38459004 PMC10923903

[B241] Ying S. Wang J. Gong L. Wang J. Sun G. (2023). The steady-state maintenance mechanism of peroxisomes and membrane contact sites. *Prog. Biochem. Biophys.* 50 704–713. 10.16476/j.pibb.2022.0547

[B242] Yu D.-D. Wang J.-M. Han L. Du X.-Y. Chao L.-Q. Zhang H.-H. (2024). Mechanism of acupuncture and moxibustion in ameliorating liver injury induced by cisplatin by regulating IRE-1 signaling pathway. *Zhen Ci Yan Jiu* 49 686–692. 10.13702/j.1000-0607.20240125 39020486

[B243] Yu S. Zeng Y. Ruan C. Bai L. Liang Z. (2023). Protective effects of brain and muscle ARNT-like gene 1 on oxidized low-density lipoprotein-induced human brain microvascular endothelial cell injury by alleviating ferroptosis. *Hum. Exp. Toxicol.* 42:9603271231184630. 10.1177/09603271231184630 37343012

[B244] Yu W. Li Y. Hu J. Wu J. Huang Y. (2022). A study on the pathogenesis of vascular cognitive impairment and dementia: The chronic cerebral hypoperfusion hypothesis. *J. Clin. Med.* 11:4742. 10.3390/jcm11164742 36012981 PMC9409771

[B245] Zhang C. Zheng J. Yu X. Kuang B. Dai X. Zheng L. (2024). “Baihui” (DU20)-penetrating “Qubin” (GB7) acupuncture on blood–brain barrier integrity in rat intracerebral hemorrhage models via the RhoA/ROCK II/MLC 2 signaling pathway. *Anim. Models Exp. Med.* 7 740–757. 10.1002/ame2.12374 38379356 PMC11528382

[B246] Zhang J. Cai W. Wei X. Shi Y. Zhang K. Hu C. (2023). Moxibustion ameliorates cerebral ischemia-reperfusion injury by regulating ferroptosis in rats. *Clin. Exp. Pharmacol. Physiol.* 50 779–788. 10.1111/1440-1681.13801 37417429

[B247] Zhang M. Xv G.-H. Wang W.-X. Meng D.-J. Ji Y. (2017). Electroacupuncture improves cognitive deficits and activates PPAR-γ in a rat model of Alzheimer’s disease. *Acupunc. Med. J. Br. Med. Acupunc. Soc.* 35 44–51. 10.1136/acupmed-2015-010972 27401747

[B248] Zhang W. Liu Y. Liao Y. Zhu C. Zou Z. (2024). GPX4, ferroptosis, and diseases. *Biomed. Pharmacother.* 174:116512. 10.1016/j.biopha.2024.116512 38574617

[B249] Zhang X. Wu B. Nie K. Jia Y. Yu J. (2014). Effects of acupuncture on declined cerebral blood flow, impaired mitochondrial respiratory function and oxidative stress in multi-infarct dementia rats. *Neurochem. Int.* 65 23–29. 10.1016/j.neuint.2013.12.004 24361538

[B250] Zhang X. Zheng H. Peiyan H. Mengning Y. Zhihui Z. Guangxia N. I. (2024). Mechanism of acupuncture in attenuating cerebral ischaemia-reperfusion injury based on nuclear receptor coactivator 4 mediated ferritinophagy. *J. Traditional Chin. Med.* 44 345–352. 10.19852/j.cnki.jtcm.20240203.006 38504540 PMC10927404

[B251] Zhao Y. Zhou B. Zhang G. Xu S. Yang J. Deng S. (2022). The effect of acupuncture on oxidative stress: A systematic review and meta-analysis of animal models. *PLoS One* 17:e0271098. 10.1371/journal.pone.0271098 36084019 PMC9462787

[B252] Zhou L. Han S. Guo J. Qiu T. Zhou J. Shen L. (2022). Ferroptosis-A new dawn in the treatment of organ ischemia-reperfusion injury. *Cells* 11:3653. 10.3390/cells11223653 36429080 PMC9688314

[B253] Zhou M. Xu K. Ge J. Luo X. Wu M. Wang N. (2024). Targeting ferroptosis in Parkinson’s Disease: Mechanisms and emerging therapeutic strategies. *Int. J. Mol. Sci.* 25:13042. 10.3390/ijms252313042 39684753 PMC11641825

[B254] Zhu K. Cai Y. Lan L. Luo N. (2024). Tumor metabolic reprogramming and ferroptosis: The impact of glucose, protein, and lipid metabolism. *Int. J. Mol. Sci.* 25:24. 10.3390/ijms252413413 39769177 PMC11676715

[B255] Zhu W. Dong J. Han Y. (2024). Electroacupuncture downregulating neuronal ferroptosis in MCAO/R rats by activating Nrf2/SLC7A11/GPX4 Axis. *Neurochem. Res.* 49 2105–2119. 10.1007/s11064-024-04185-x 38819696 PMC11233380

[B256] Zilka O. Shah R. Li B. Angeli J. P. F. Griesser M. Conrad M. (2017). *On the Mechanism of Cytoprotection by Ferrostatin-1 and Liproxstatin-1 and the Role of Lipid Peroxidation in Ferroptotic Cell Death (world) [Research-article].* Washington, DC: ACS Publications, 10.1021/acscentsci.7b00028 PMC5364454 28386601

[B257] Zou Y. Henry W. S. Ricq E. L. Graham E. T. Phadnis V. V. Maretich P. (2020a). Plasticity of ether lipids promotes ferroptosis susceptibility and evasion. *Nature* 585 603–608. 10.1038/s41586-020-2732-8 32939090 PMC8051864

[B258] Zou Y. Li H. Graham E. T. Deik A. A. Eaton J. K. Wang W. (2020b). Cytochrome P450 oxidoreductase contributes to phospholipid peroxidation in ferroptosis. *Nat. Chem. Biol.* 16 302–309. 10.1038/s41589-020-0472-6 32080622 PMC7353921

[B259] Zuo T. Jianbin Z. Luqi H. (2017). Protective effect of electroacupuncture on neurons autophagy in perfusion period of cerebral ischemia. *Neurosci. Lett.* 661 41–45. 10.1016/j.neulet.2017.06.043 28663053

